# A comprehensive analysis of COVID-19 vaccination behavior: The influence of religion, information sources, political leanings, and demographic factors

**DOI:** 10.1371/journal.pone.0323815

**Published:** 2025-05-21

**Authors:** Defne Över, Emilce Santana, Ernesto F. L. Amaral, Chaitanya Lakkimsetti, Anna Estelle Kelley, Dulce Angelica Espinoza

**Affiliations:** Department of Sociology, Texas A&M University, College Station, Texas, United States of America; Georgetown University Medical Center, UNITED STATES OF AMERICA

## Abstract

The COVID-19 pandemic highlighted the crucial role of vaccines in controlling the virus. Despite their effectiveness, however, vaccine hesitancy remained a challenge, particularly within certain population groups. This multi-disciplinary study investigates the diverse socio-demographic factors influencing COVID-19 vaccination decisions in the United States. Through a nationally representative survey of 5,240 people, the research explores the interplay of information sources, religious beliefs, political party, and demographic characteristics of the respondents. Our findings reveal associations of main sources of information with vaccination likelihood, with the Centers for Disease Control and Prevention demonstrating the highest association with full vaccination. Religious beliefs are significant determinants, with Evangelical Protestants exhibiting the lowest vaccination rates. We also highlight the intricate relationship between political leanings and vaccination behavior, emphasizing higher levels of vaccination among Democrats. Demographic variables, including age, education, gender, and race/ethnicity, also play pivotal roles, exposing disparities in vaccination access and decisions. In particular, older individuals and those with higher levels of education show a greater inclination to achieve full vaccination, while women and African Americans are less likely to attain complete vaccination. Lastly, while major ethnoracial groups seem to respond to different sources of information similarly, there are also nuanced differences, such as Asians being especially likely to be fully vaccinated if they depend on the CDC or other health sources while more disadvantaged groups seem less responsive to these sources. Overall, this research provides a comprehensive analysis of the nuanced factors shaping vaccination behavior. It contributes valuable knowledge to public health strategies, emphasizing the need for targeted communication campaigns tailored to diverse communities.

## Introduction

During the COVID-19 pandemic, vaccines emerged as a highly effective method of providing protection against the wild type SARS-CoV-2 [1,2]. Medical reports published during the pandemic established the role of vaccines in limiting the spread of the virus [3]. COVID-19 vaccines thus provided a major opportunity to return to normalcy if a significant number of people got vaccinated. Yet, vaccination refusal and hesitancy within communities remained a major impediment on the road to normalcy [4]. In many places in the United States where COVID-19 vaccines were widely accessible, vaccination rates remained below the desired threshold, even when infection rates in the given community were high [5–7]. Focusing on the case of the United States, this mixed method study examines key factors influencing vaccination decision-making during the COVID-19 pandemic.

Individuals adopt different behaviors toward vaccination. Some are complete acceptors, others total refusers and there are also hesitant individuals who may refuse some vaccines, but agree to others; delay vaccines; or accept vaccines but are unsure of doing so [8]. Researchers recognize that factors influencing vaccine behavior vary depending on time, place, and the type of vaccine [9–11], and trace the variation in vaccine acceptance to reasons such as lack of trust in vaccine administrators; individual and group level perceptions of the vaccine; and the characteristics of the vaccine [12,13].

Research on other vaccination programs, e.g., World Health Organization (WHO)’s Expanded Program on Immunization in India, the state-mandated childhood vaccines in the US, and human papillomavirus (HPV) vaccines in the US, for instance, has shown that viewing vaccination as part of a political agenda, perceiving health as a matter of individual choice rather than public decision making, personalization of risks associated with vaccination, presence of misinformation and organized vaccine resistance in one’s community increased the likelihood of vaccine hesitancy and refusal in a given community [9,14–18]. Research on the swine influenza vaccine in the UK has shown that resourceless and marginalized peoples and disadvantaged minority groups are less likely to get vaccinated [19].

While situations under which we observed COVID-19 vaccine hesitancy vastly differ from vaccine hesitancy for children’s diseases, HPV, or influenza [20], there are parallels in ambiguities experienced in relation to vaccine safety, and lack of trust in the state, and public health institutions in certain communities, showing that vaccination is not only an individual but also a community decision. Recognizing the impact of community membership on individuals’ vaccination decisions, this article examines how people with different socioeconomic, demographic, political, and religious backgrounds responded to COVID-19 vaccination campaigns in the US. Specifically, we inquire into the connections between beliefs (religious, and political), sources of information (religious leaders, health professionals, the media, political actors), community characteristics (age, level of education, race/ethnicity, gender), and individuals’ vaccine behaviors in the COVID-19 pandemic.

The study is based on a nationally representative survey with national quotas for age, sex, race/ethnicity, and political party affiliation, distributed to 5,240 adults in the United States. In what follows, we present the scholarly literature on vaccination behavior in the US during the COVID-19 pandemic, our data collection and analysis methods, and the results of our analysis. We conclude the article with a discussion on the implications of our findings for future vaccination campaigns, in particular for communication strategies aimed at increasing vaccination uptake.

### Vaccine behavior in the US during the COVID-19 pandemic

The COVID-19 pandemic has affected communities across the US disproportionately. In Texas, for example, with 40% Hispanic and 40% White population, the death toll in the Hispanic community was three times that of the White population when adjusted for age [21]. Despite the higher risks encountered by minority groups, research has found that there exist racial and ethnic differences in vaccine hesitancy and refusal [22–25], with minoritized groups reporting higher levels of vaccine hesitancy [26–32]. African Americans and Hispanics, in particular, have led racial and ethnic groups in vaccine hesitancy while Whites had the highest share of eligible people who received vaccination. For example, while the Hispanic population had higher chances of morbidity, hospitalization, and death due to COVID-19, only 25% of Hispanics had received a vaccine shot in Texas compared to 50% of Whites by March 15, 2021 [5]. In the same period, over 30% of Hispanic adults wanted to “wait and see” how the vaccine works for other people, and 20% refused to vaccinate unless required to do so [6]. Some researchers posited institutional trust as a reason for lower levels of vaccination among minority groups–particularly African Americans [23,25,29,33,34]. Accordingly, African Americans were more likely to refuse vaccination than Whites for the sole reason that they do not trust COVID vaccine to be safe or effective [31,33,35]. This trend in vaccine attitudes remained despite the fact that African Americans have widely been found more likely to be at risk of and express concern for the virus than Whites [26,29].

Studies looking at the relationship between sex and gender and vaccine uptake have, on the other hand, shown mixed results. Research conducted early in the pandemic showed that women expressed greater vaccine hesitancy than men [27,36,37]. A scoping review of the literature on vaccine uptake in the first two years of the pandemic showed that while men did tend to have greater vaccine uptake intentions, there were limited differences in actual uptake [38]. Studies conducted in later years of the pandemic continued to show greater vaccine hesitancy among women than men, but data collected at vaccination sites indicate that women have higher vaccination rates than men [25].

In terms of age, older adults were more likely than younger adults to express willingness to be vaccinated, and unlike gender, this was reflected in vaccine uptake [25,27,28,39,40]. Young adults were also more likely to report being unwilling or undecided about getting the vaccine [27]. A survey conducted at a mid-sized public university in Connecticut, for example, revealed that about 30% of college students indicated vaccine refusal, and about 20% of college students were unsure about whether or not to get vaccinated once they were eligible [7]. At the same time, those with higher levels of education were more likely to report higher levels of COVID-19 vaccine acceptance. Early in the pandemic, those with college or graduate degrees were more likely to report willingness to receive the vaccine than those with lower levels of educational attainment [27,30,37]. Those with higher levels of education were also more likely to have actually gotten the vaccine once it became available [39]. Additionally, those with higher income levels had higher levels of vaccine acceptance and trust, while those with lower income levels were more likely to report vaccine hesitancy [25,28].

To explain the variation in vaccine uptake across groups, some scholars approached vaccination behavior as a political decision. Exploring the effect of partisanship, conservatism, and Trumpism as factors driving vaccine refusal or hesitancy, they found that political conservatism is significantly associated with greater skepticism toward COVID-19 in general [41,42]. Researchers further indicated that being more politically conservative is a consistently negative predictor of vaccination likelihood [25,43]. While some [43] have argued that the partisan divide in vaccine hesitancy has increased over time, others [25] have shown that political conservatism became a consistent significant negative predictor of trust in the vaccine starting in November 2020, when the U.S. elected Joe Biden as President. Others confirmed these findings by showing that democrats are more likely to have gotten vaccinated against COVID-19 than other adults [39]; that increased Republican affiliation correlated with lower vaccination rates [44]; and that there is a negative relationship between Trump support and COVID-19 vaccination rate [45,46].

Further research into the relationship between political identity and vaccine uptake explored how political identity interacted with other factors in predicting attitudes towards the COVID-19 vaccine. Researchers have found that support for Trump and conservatism were strong predictors of negative attitudes toward vaccination [47]. They have also found that type of religious identification, in particular identification with Christian nationalism, was significant for vaccine hesitancy [42]. These researchers also suggested that Christian nationalism was a strong predictor that individuals would be less likely to engage in precautions such as avoiding crowded venues, wearing a mask, and other protective public health practices. Others have similarly proposed that Christian nationalism as a pervasive ideology that rejects scientific authority and promotes allegiance to conservative political leaders was one of the two strongest predictors of anti-vaccine attitudes, stronger than political or religious characteristics considered separately [48].

Digging deeper into the variation in vaccination behaviors of racial, ethnic, gender, age and political communities, scholars have pointed at information sources as a significant factor driving vaccination decisions [54]. Accordingly, social media platforms such as Facebook and Twitter contributed to the unregulated spread of misinformation about COVID-19 [49]. Interaction with negative tweets about COVID-19, in particular, was associated with expressions of lack of confidence in vaccine safety, distrust in governments and experts, and widespread misinformation or rumors [50]. Scholars also found that conservative media outlets, along with political and thought leaders, played a crucial role in downplaying the virus and discouraging adherence to recommended public health guidelines [45,49,51]. Followers of conservative news networks such as *Fox News*, those who cited Trump, his task force, and personal and community networks as their favored COVID-19 news sources were found to be far less likely than those who relied on other information sources to have received at least one shot of the vaccine. Similarly, those who reported seeking information from religious leaders were less likely to be vaccinated [52]. In contrast, those that got information from official government and health agencies, healthcare providers as well as those who followed more mainstream news sources were more likely to take the COVID-19 pandemic seriously [53]. For example, those that got COVID-19 related information from *The New York Times* and commercial news sources like *CNN* or *MSNBC* reported greater concern about contracting COVID-19 [54].

Building on existing research, the current study examines the impact of race and ethnicity, gender, age, political and religious beliefs, and information sources on COVID-19 vaccination decisions. It extends these studies by investigating how individuals’ racial and ethnic backgrounds interact with their reliance on specific information sources to shape vaccination choices. Unlike prior research, which typically focuses on specific ethnic and racial groups’ vaccination decisions, by relying on a nationally representative sample, this study explores whether different racial and ethnic groups rely on different information sources and whether this reliance influences their vaccination behavior in varying ways. The study thus aims to offer empirically grounded strategies for the use of information sources in the communication of public health messages to specific communities.

Our main exploratory hypotheses are the following:

Hypothesis 1: More vulnerable populations (e.g., ethno-racial minorities, women, elderly, lower-educated individuals) face obstacles to follow public health practices due to several factors (e.g., being overrepresented in essential jobs, having less access to government benefits) and are less likely to be vaccinated against COVID-19.

Hypothesis 2: Individuals who have conservative political and religious beliefs are less likely to follow these practices and to be vaccinated against COVID-19, compared to individuals who have liberal political and religious beliefs.

Hypothesis 3: Individuals who receive COVID-19 related information primarily from their informal networks (family, employer, friends) are less likely to be vaccinated against COVID-19, compared to individuals who primarily follow health experts as information sources.

### Methods

This research project is registered at Texas A&M University Institutional Review Board with the IRB ID: IRB2020-0722D. We started this project with a qualitative pilot study undertaken in Brazos County, Texas in 2021. For the pilot study, we conducted interviews via phone with 44 adults aged 18 and above. The research team obtained verbal consent from the interviewees using an IRB-approved consent script. During each interview session, participants were prompted to answer 31 open-ended questions about their racial, religious, political, socio-economic characteristics, their lived experiences of the pandemic, and their strategies of coping with COVID-19 including social distancing and mask wearing. The interviews highlighted the variation in the level of peer pressure, lived experiences of COVID-19 pandemic, and belief in science across age and gender groups, ethnic and racial communities, and socio-economic status. The interviews pointed at a major variation in the sources that people used to gather information on COVID-19. While 17.7% of interviewees mentioned using news apps as main sources of information related to the COVID-19 pandemic, 24.4% claimed to have gathered information primarily from their friends and family, and 57.7% used social media as a news source of information. The interviews also revealed that strategies of coping with the conditions created by COVID-19 along with the motivations behind these strategies varied significantly among the interviewees.

Based on the findings of the qualitative pilot study, we designed a nationally representative survey with questions about factors driving individuals’ COVID-19 vaccine decisions in the United States. At the beginning of the survey, respondents read out the informed consent document. If they agreed with the document, the online survey was administered. The survey was fielded solely online and was distributed between September 5, 2023, and September 9, 2023, via the Prime Panel service from CloudResearch. Prime Panels are an “aggregation of opt-in market research panels” [55]. More specifically, multiple suppliers bid to CloudResearch for the opportunity to supply respondents to the specified samples requested by different research teams [56]. The benefit of this approach is that our research team was able to sample subpopulations that may be difficult to reach [55,56]. CloudResearch Prime Panels are an advancement upon other data collection softwares, such as Amazon Mechanical Turk, because it is more demographically targeted and thus able to be more representative. Participants are compensated in a number of ways, based on the platform through which the individual participates. It includes compensation methods such as reward points, gift cards, or cash. Our team was able to assign sampling quotas to ensure a representative sample [55,56]. Our team selected respondents according to national quotas available in the CloudResearch Prime Panel tool, according to the US population distributed by age, sex, race, ethnicity, and political party. Respondents were provided by the suppliers who submitted their bids to this research project with Cloud Research. The quota for age considered the following groups: 18–29 (21.62% of respondents), 30–44 (26.55%), 45–59 (24.76%), and 60–99 (27.07%). The quota for sex, considered female (52.00%), and male (48.00%). For race, the quota selected respondents to reach this distribution: White (75.60%), African American (11.80%), Asian (4.73%), American Indian/Alaska Native (1.79%), and other races (6.10%). The quota for ethnicity utilized these two groups: Hispanic (17.44%) and not Hispanic (82.58%). For political party affiliation, the quota selected respondents according to three groups: Democrat (38.85%), Republican (34.31%), and Independent (26.84%). CloudResearch received a total of 5,246 respondents distributed in the quotas listed above. These respondents answered 100% of the questions and there were no duplicates. This data collection strategy does not provide a response rate as in traditional data collection methods. For this specific paper, we kept responses from respondents who identified as either male or female in the assigned sex at birth category, which resulted in dropping six responses, and a total of 5,240 respondents.

The survey captured respondents’ vaccine behaviors alongside their community characteristics, the sources that they have received information from, their perceptions on a variety of issues ranging from politics to science and vaccination, and their lived experiences of COVID-19 pandemic. Our questionnaire had a total of 88 questions related to 12 blocks: (1) COVID-19 experience; (2) COVID-19 vaccine behavior; (3) views on science, vaccines, and the COVID-19 pandemic; (4) sources of information about the COVID-19 pandemic and vaccines; (5) misinformation and fake news about the COVID-19 pandemic; (6) gender and domestic work; (7) information about current residence; (8) demographic characteristics; (9) religious beliefs; (10) political background; (11) main occupation; and (12) income. More specifically, we collected information on behavioral, socioeconomic, and demographic measures such as race/ethnicity, social class, gender, educational attainment, religion, occupation, health insurance, sick leave, political ideology, migration status, membership to community associations, and consumption of media sources.

### Statistical analysis

To test our hypotheses about likelihood of being vaccinated against COVID-19, we selected a variable about vaccination as the dependent variable for a logistic regression model. Out of 5,240 individuals in our sample, 3,644 were fully vaccinated, 389 were partially vaccinated, and 1,207 were unvaccinated. For this study, we combined individuals who were partially vaccinated and those who were not vaccinated for COVID-19 to compare with those who were fully vaccinated.

We also tested different specifications of the dependent variable. For instance, we estimated logistic regression models by combining fully and partially vaccinated individuals to compare with those not vaccinated. Additionally, we estimated a multinomial logistic regression that utilized the original variable with three categories related to COVID-19 vaccination by estimating in the same model comparisons between partially vaccinated and fully vaccinated, as well as between unvaccinated and fully vaccinated. These additional specifications were not different from the analysis provided in this study.

Our overall rationale is that at that time of our survey in September 2023, individuals who were not fully vaccinated made the decision to be either partially vaccinated or unvaccinated. Thus, we focus the analysis on logistic regressions that estimate the chances of individuals being fully vaccinated, controlling for a series of independent variables.

We test Hypothesis 1 about more vulnerable populations being less likely to be vaccinated against COVID-19 with a series of sociodemographic variables: Race/ethnicity, assigned sex at birth; age groups; and education levels. This way we could verify if ethno-racial minorities, women, older individuals, and individuals with lower education levels are less likely to be vaccinated. The race/ethnicity groups utilized in our analysis are: (1) Non-Hispanic White; (2) Hispanic; (3) Non-Hispanic African American; (4) Non-Hispanic Asian; (5) Other race/ethnicity or two or more races/ethnicities. For assigned sex at birth, a total of six individuals reported an undetermined sex. For the current analysis, we only include individuals who reported male or female as the assigned sex at birth. Our age groups (18–29, 30–39, 40–49, 50–64, 65–74, 75+) follow the standard utilized by the CDC when reporting COVID-19 indicators. We classified the sample in the following education groups: (1) Less than high school; (2) High school; (3) Some college; (4) Associate degree; (5) Bachelor’s degree; and (6) Graduate degree.

Our Hypothesis 2 states that individuals with more conservative political and religious beliefs are less likely to be vaccinated against COVID-19. In order to test this hypothesis, we included disaggregated categories for political parties and religious beliefs to explore detailed associations of these variables with COVID-19 vaccination. Political parties include these categories: (1) Strong Democrat; (2) Democrat; (3) Independent; (4) Republican; (5) Strong Republican; and (6) other parties. Religious preference includes the following categories: (1) Evangelical Protestant; (2) Other Protestant; (3) Catholic; (4) Other; (5) Agnostic or Atheist; and (6) Nothing in particular.

As a strategy to test Hypothesis 3 that individuals who follow family, friends and employer as their primary source of information on COVID-19 are less likely to be vaccinated against COVID-19, we included the main source of information about COVID-19 vaccines as an independent variable. This question has the following categories: (1) Centers for Disease Control and Prevention (CDC); (2) Employer; (3) Family and friends; (4) Food and Drug Administration (FDA); (5) Health insurers; (6) Hospital system websites (e.g., Kaiser Permanente); (7) Local health officials; (8) News sources (e.g., television, internet, and radio); (9) Nurses; (10) Pharmacists; (11) Primary care providers; (12) Professional organizations; (13) Religious leaders; (14) State health departments; (15) Online publishers of medical information (e.g., WebMD or Mayo Clinic); (16) Social media (e.g., Facebook, Twitter, Instagram); (17) Union leaders; and (18) Other sources. We grouped these sources of information based on how related they are to each other, as well as based on the percentage of respondents who were fully vaccinated for COVID-19 in each category. Based on these criteria, we simplified this variable into five categories: (1) CDC, which had 83% of respondents fully vaccinated; (2) Other health sources, which includes categories that had between 53% and 73% of respondents fully vaccinated: FDA, health insurers, hospital system websites, local health officials, nurses, pharmacists, primary care providers, state health departments, online medical publishers; (3) Employer (58% of respondents fully vaccinated) and family and friends (47%) became one category; (4) News sources (66%) and professional organizations (73%) were combined; (5) Other sources, which includes categories that had between 25% and 40% of respondents fully vaccinated: Religious leaders, social media, union leaders, and other sources.

We examined the percentage distribution of respondents by all variables included in our analysis, as well as cross-tabulations between all independent variables and the dependent variable: Whether individuals are fully vaccinated for COVID-19 (Table 1). Then, we estimated a series of bivariate logistic regression models to analyze associations of each independent variable with the dependent variable (Table 2). Additionally, we estimated a multivariate logistic regression including all independent variables predicting the dependent variable (Table 3).

**Table 1:** Percentage distribution of respondents by variables of interest and cross-tabulation with COVID-19 vaccination status, 2023.

Variables	Column %	Fully vaccinated (row %)	Not fully vaccinated (row %)	Sample size	Association with vaccination
**COVID-19 vaccination status**	–––	69.54	30.46	5,240	–––
**Sex**					Chi square
Male	47.50	73.72	26.28	2,489	(df = 1):
Female	52.50	65.76	34.24	2,751	39.15^***^
**Age group**					Chi square
18–29	19.39	59.55	40.45	1,016	(df = 5):
30–39	19.27	65.45	34.55	1,010	177.66^***^
40–49	17.27	63.76	36.24	905	
50–64	22.50	73.20	26.80	1,179	
65–74	14.62	81.59	18.41	766	
75+	6.95	85.99	14.01	364	
**Education group**					Chi square
Less than high school	3.17	40.36	59.64	166	(df = 5):
High school	25.13	56.04	43.96	1,317	394.77^***^
Some college	21.09	64.80	35.20	1,105	
Associate	12.00	68.36	31.64	629	
Bachelor	23.51	80.93	19.07	1,232	
Graduate	15.10	87.99	12.01	791	
**Political party**					Chi square
Strong Democrat	25.31	86.65	13.35	1,326	(df = 5):
Democrat	18.97	76.06	23.94	994	343.03^***^
Independent	20.13	61.14	38.86	1,055	
Republican	16.95	59.01	40.99	888	
Strong Republican	17.54	58.54	41.46	919	
Other party	1.11	55.17	44.83	58	
**Religious group**					Chi square
Evangelical Protestant	14.58	62.83	37.17	764	(df = 5):
Other Protestant	15.59	71.11	28.89	817	167.32^***^
Catholic	24.92	76.80	23.20	1,306	
Other	5.34	85.36	14.64	280	
Agnostic/Atheist	9.71	78.59	21.41	509	
Nothing in particular	29.85	60.17	39.83	1,564	
**Race/ethnicity**					Chi square
White	68.07	70.31	29.69	3,567	(df = 4):
Hispanic	13.21	68.35	31.65	692	37.61^***^
African American	11.15	63.01	36.99	584	
Asian	3.53	85.41	14.59	185	
Other race/ethnicity	4.05	64.62	35.38	212	
**Main source of information about vaccines**					Chi square
Centers for Disease Control and Prevention (CDC)	30.84	83.35	16.65	1,616	(df = 4):
Other health sources	49.71	68.06	31.94	2,605	392.84^***^
Employer, family, and friends	9.26	49.28	50.72	485	
News sources and professional organizations	6.51	67.16	32.84	341	
Other sources	3.68	29.02	70.98	193	

Note:

* Significant at p < 0.1;

** Significant at p < 0.05;

*** Significant at p < 0.01.

Source: 2023 Survey about Effective Communication Strategies During the COVID-19 Pandemic.

**Table 2:** Odds ratios and exponentials of standard errors from bivariate logistic regression models predicting whether individuals are fully vaccinated for COVID-19, 2023.

Independent variables	Odds ratios	Exponential of standard errors	Pseudo R^2^
**Sex**			0.006
Male	ref.		
Female	0.684^***^	0.042	
**Age group**			0.029
18–29	0.777^***^	0.072	
30–39	ref.		
40–49	0.929	0.089	
50–64	1.442^***^	0.135	
65–74	2.340^***^	0.268	
75+	3.240^***^	0.534	
**Education group**			0.064
Less than high school	0.531^***^	0.089	
High school	ref.		
Some college	1.444^***^	0.121	
Associate	1.695^***^	0.173	
Bachelor	3.329^***^	0.304	
Graduate	5.748^***^	0.705	
**Political party**			0.057
Strong Democrat	ref.		
Democrat	0.489^***^	0.054	
Independent	0.242^***^	0.025	
Republican	0.222^***^	0.023	
Strong Republican	0.218^***^	0.023	
Other party	0.190^***^	0.052	
**Religious group**			0.027
Evangelical Protestants	ref.		
Other Protestant	1.457^***^	0.157	
Catholic	1.959^***^	0.195	
Other	3.449^***^	0.638	
Agnostic/Atheist	2.171^***^	0.285	
Nothing in particular	0.894	0.081	
**Race/ethnicity**			0.006
White	ref.		
Hispanic	0.912	0.082	
African American	0.719^***^	0.067	
Asian	2.471^***^	0.522	
Other race/ethnicity	0.771^*^	0.114	
**Main source of information about COVID-19 vaccines**			0.060
Centers for Disease Control and Prevention (CDC)	ref.		
Other health sources	0.426^***^	0.034	
Employer, family, and friends	0.194^***^	0.022	
News sources and professional organizations	0.408^***^	0.054	
Other sources	0.082^***^	0.014	

Note: All models have a sample size of 5,240 respondents. Constant terms of each model are not reported.

* Significant at p < 0.1;

** Significant at p < 0.05;

*** Significant at p < 0.01.

Source: 2023 Survey about Effective Communication Strategies During the COVID-19 Pandemic.

**Table 3:** Odds ratios and exponentials of standard errors from a multivariate logistic regression model predicting whether individuals are fully vaccinated for COVID-19, 2023.

Independent variables	Odds ratios	Exponential of standard errors
Constant	4.136^***^	0.707
**Sex**		
Male	ref.	
Female	0.747^***^	0.051
**Age group**		
18–29	0.978	0.101
30–39	ref.	
40–49	1.023	0.110
50–64	1.693^***^	0.179
65–74	2.345^***^	0.301
75+	3.245^***^	0.590
**Education group**		
Less than high school	0.584^***^	0.106
High school	ref.	
Some college	1.222^**^	0.112
Associate	1.470^***^	0.164
Bachelor	2.551^***^	0.257
Graduate	3.722^***^	0.501
**Political party**		
Strong Democrat	ref.	
Democrat	0.583^***^	0.069
Independent	0.365^***^	0.041
Republican	0.292^***^	0.034
Strong Republican	0.297^***^	0.035
Other party	0.305^***^	0.097
**Religious group**		
Evangelical Protestants	ref.	
Other Protestant	1.177	0.142
Catholic	1.567^***^	0.175
Other	1.785^***^	0.369
Agnostic/Atheist	1.652^***^	0.250
Nothing in particular	1.050	0.111
**Race/ethnicity**		
White	ref.	
Hispanic	1.163	0.120
African American	0.827^*^	0.089
Asian	2.186^***^	0.497
Other race/ethnicity	0.886	0.149
**Main source of information about COVID-19 vaccines**		
Centers for Disease Control and Prevention (CDC)	ref.	
Other health sources	0.551^***^	0.047
Employer, family, and friends	0.339^***^	0.042
News sources and professional organizations	0.477^***^	0.070
Other sources	0.111^***^	0.021
Likelihood Ratio Chi-squared Test (df = 29)	1,120.84^***^	
Pseudo R-squared	0.174	
Observations	5,240	

Note:

* Significant at p < 0.1;

** Significant at p < 0.05;

*** Significant at p < 0.01.

Source: 2023 Survey about Effective Communication Strategies During the COVID-19 Pandemic.

Finally, we verified that the percentage of respondents fully vaccinated by the main information source and race/ethnicity vary considerably (Table 4). In order to statistically test these variations, we estimated models to evaluate interaction effects between race/ethnicity and information source for COVID-19 vaccination. Table 5 includes these interactions as a set of dummies. Table 6 illustrates results from separate models for each race/ethnicity, which is equivalent to interacting race/ethnicity with all independent variables. The sample sizes are small for some sub-groups of respondents by the main source of information about vaccines and race/ethnicity (e.g., Non-Hispanic Asian and other race/ethnicity). Due to this limitation, for models on Table 6, we focus the analysis on Non-Hispanic White, Hispanic, and Non-Hispanic African American.

**Table 4:** Percentage distribution of respondents by source of information about COVID-19 vaccines and race/ethnicity, as well as percentage of those fully vaccinated in each sub-group, 2023.

Main source of information about COVID-19 vaccines	Race/ethnicity					
	White	Hispanic	African American	Asian	Other	Total
**Centers for Disease Control and Prevention (CDC)**						
Sample size	1,112	196	172	73	63	1,616
Column percentage	31.17	28.32	29.45	39.46	29.72	30.84
Percentage fully vaccinated	84.17	82.14	76.74	95.89	76.19	83.35
**Other health sources**						
Sample size	1,790	360	274	83	98	2,605
Column percentage	50.18	52.02	46.92	44.86	46.23	49.71
Percentage fully vaccinated	69.11	64.17	61.31	85.54	67.35	68.06
**Employer, family, and friends**						
Sample size	320	76	63	5	21	485
Column percentage	8.97	10.98	10.79	2.70	9.91	9.26
Percentage fully vaccinated	49.38	59.21	41.27	20.00	42.86	49.28
**News sources and professional organizations**						
Sample size	219	39	50	19	14	341
Column percentage	6.14	5.64	8.56	10.27	6.60	6.51
Percentage fully vaccinated	64.84	84.62	68.00	68.42	50.00	67.16
**Other sources**						
Sample size	126	21	25	5	16	193
Column percentage	3.53	3.03	4.28	2.70	7.55	3.68
Percentage fully vaccinated	27.78	14.29	32.00	60.00	43.75	29.02
**Total**						
Sample size	3,567	692	584	185	212	5,240
Column percentage	100.00	100.00	100.00	100.00	100.00	100.00
Percentage fully vaccinated	70.31	68.35	63.01	85.41	64.62	69.54

Note: Percentage of respondents fully vaccinated for COVID-19 are reported between parentheses.

Source: 2023 Survey about Effective Communication Strategies During the COVID-19 Pandemic.

**Table 5:** Odds ratios and exponentials of standard errors from a multivariate logistic regression model predicting whether individuals are fully vaccinated for COVID-19, 2023.

Independent variables	Odds ratios	Exponential of standard errors
**Race/ethnicity interacted with** **main source of information about COVID-19 vaccines**		
**White**		
Centers for Disease Control and Prevention (CDC)	ref.	
Other health sources	0.545^***^	0.057
Employer, family, and friends	0.313^***^	0.048
News sources and professional organizations	0.367^***^	0.067
Other sources	0.101^***^	0.024
**Hispanic**		
Centers for Disease Control and Prevention (CDC)	1.054	0.228
Other health sources	0.550^***^	0.083
Employer, family, and friends	0.562^**^	0.150
News sources and professional organizations	1.431	0.677
Other sources	0.057^***^	0.036
**African American**		
Centers for Disease Control and Prevention (CDC)	0.720	0.153
Other health sources	0.423^***^	0.069
Employer, family, and friends	0.253^***^	0.073
News sources and professional organizations	0.609	0.204
Other sources	0.113^***^	0.051
**Asian**		
Centers for Disease Control and Prevention (CDC)	4.137^**^	2.507
Other health sources	1.357	0.459
Employer, family, and friends	0.072^**^	0.081
News sources and professional organizations	0.616	0.325
Other sources	0.254	0.252
**Other race/ethnicity**		
Centers for Disease Control and Prevention (CDC)	0.634	0.209
Other health sources	0.488^***^	0.122
Employer, family, and friends	0.272^***^	0.131
News sources and professional organizations	0.379^*^	0.216
Other sources	0.221^***^	0.121
Likelihood Ratio Chi-squared Test (df = 45)	1,148.55^***^	
Pseudo R-squared	0.178	
Observations	5,240	

Note: This model is controlled for sex, age group, education group, political party, and religious group.

* Significant at p < 0.1;

** Significant at p < 0.05;

*** Significant at p < 0.01.

Source: 2023 Survey about Effective Communication Strategies During the COVID-19 Pandemic.

**Table 6:** Odds ratios and exponentials of standard errors from a multivariate logistic regression model for each race/ethnicity category predicting whether individuals are fully vaccinated for COVID-19, 2023.

Independent Variables	Race/ethnicity				
	White	Hispanic	African American	Asian	Other
Constant	3.962^***^	17.372^***^	2.744^***^	5.928	1.742
	(0.847)	(9.615)	(1.048)	(10.469)	(1.497)
**Sex**					
Male	ref.	ref.	ref.	ref.	ref.
Female	0.708^***^	0.650^**^	0.843	1.284	0.922
	(0.061)	(0.125)	(0.163)	(0.716)	(0.327)
**Age group**					
18–29	0.956	1.097	0.853	2.767	0.929
	(0.128)	(0.283)	(0.236)	(2.179)	(0.516)
30–39	ref.	ref.	ref.	ref.	ref.
40–49	0.986	0.863	1.214	1.922	2.461
	(0.134)	(0.252)	(0.344)	(1.895)	(1.530)
50–64	1.732^***^	1.214	1.839^**^	3.669	1.974
	(0.229)	(0.352)	(0.540)	(3.291)	(1.072)
65–74	2.672^***^	0.790	2.690^***^	5.944	1.444
	(0.422)	(0.340)	(1.022)	(6.443)	(0.801)
75+	3.512^***^	1.802	4.579^*^	0.996	2.522
	(0.734)	(1.458)	(3.629)	(1.350)	(2.010)
**Education group**					
Less than high school	0.589^**^	0.541	0.597	dropped^1^	0.152
	(0.145)	(0.235)	(0.251)		(0.179)
High school	ref.	ref.	ref.	ref.	ref.
Some college	1.178	1.439	1.121	3.052	1.617
	(0.136)	(0.354)	(0.287)	(2.544)	(0.745)
Associate	1.493^***^	1.915^**^	1.213	6.650^*^	0.918
	(0.210)	(0.583)	(0.359)	(7.327)	(0.512)
Bachelor	2.508^***^	3.396^***^	2.027^**^	8.726^**^	5.724^***^
	(0.305)	(1.071)	(0.590)	(7.850)	(3.563)
Graduate	4.133^***^	3.554^***^	4.339^***^	5.343^*^	2.498
	(0.680)	(1.467)	(2.277)	(5.260)	(1.528)
**Political party**					
Strong Democrat	ref.	ref.	ref.	ref.	ref.
Democrat	0.631^***^	0.261^***^	0.615^*^	3.356	0.776
	(0.098)	(0.097)	(0.160)	(3.714)	(0.465)
Independent	0.360^***^	0.171^***^	0.498^**^	2.525	0.362^*^
	(0.053)	(0.061)	(0.138)	(2.325)	(0.207)
Republican	0.296^***^	0.119^***^	0.398^***^	1.936	0.355^*^
	(0.044)	(0.047)	(0.127)	(1.796)	(0.204)
Strong Republican	0.274^***^	0.249^***^	0.659	0.743	0.246^**^
	(0.040)	(0.109)	(0.213)	(0.851)	(0.152)
Other party	0.267^***^	0.368	0.074^**^	dropped^1^	0.177^*^
	(0.116)	(0.275)	(0.091)		(0.174)
**Religious group**					
Evangelical Protestants	ref.	ref.	ref.	ref.	ref.
Other Protestant	1.407^**^	0.327^***^	1.027	dropped^1^	1.010
	(0.206)	(0.135)	(0.316)		(0.608)
Catholic	1.766^***^	1.046	1.189	0.123	1.667
	(0.240)	(0.349)	(0.422)	(0.177)	(1.070)
Other	2.041^***^	1.086	0.951	0.402	3.075
	(0.514)	(0.835)	(0.561)	(0.636)	(3.239)
Agnostic/Atheist	1.934^***^	0.740	1.206	0.184	2.179
	(0.349)	(0.345)	(0.710)	(0.278)	(1.709)
Nothing in particular	1.105	0.814	0.966	0.129	1.190
	(0.143)	(0.286)	(0.254)	(0.179)	(0.676)
**Main source of information about COVID-19 vaccines**					
Centers for Disease Control and Prevention (CDC)	ref.	ref.	ref.	ref.	ref.
Other health sources	0.554^***^	0.424^***^	0.590^**^	0.259^*^	0.990
	(0.059)	(0.104)	(0.140)	(0.192)	(0.439)
Employer, family, and friends	0.327^***^	0.437^**^	0.373^***^	dropped^1^	0.480
	(0.050)	(0.146)	(0.128)		(0.304)
News sources and professional organizations	0.368^***^	0.995	0.828	0.080^**^	0.626
	(0.068)	(0.518)	(0.315)	(0.079)	(0.461)
Other sources	0.103^***^	0.046^***^	0.156^***^	0.028^**^	0.438
	(0.025)	(0.032)	(0.077)	(0.040)	(0.291)
Likelihood Ratio Chi-squared Test	847.95^***^ (df = 25)	155.08^***^ (df = 25)	102.20^***^ (df = 25)	32.73^**^ (df = 21)	47.76^***^ (df = 25)
Pseudo R-squared	0.195	0.180	0.133	0.247	0.173
Observations	3,567	692	584	162	212

^1^Among Asians, the statistical software automatically dropped some individuals from the analysis, because in specific categories all individuals were fully vaccinated (predicting success perfectly). A total of 23 respondents were omitted: three respondents with less than high school; two respondents with other party affiliation; 14 respondents in other Protestant religious group; and four respondents whose main source of information about COVID-19 vaccines was from employer, family, and friends. As a result, the sample size of Asians dropped from 185 to 162 individuals.

Note: Exponential of standard errors are reported between parentheses.

* Significant at p < 0.1;

** Significant at p < 0.05;

*** Significant at p < 0.01.

Source: 2023 Survey about Effective Communication Strategies During the COVID-19 Pandemic.

We conducted a power analysis using G*Power software to determine the required sample size for a logistic regression analysis examining factors associated with being fully vaccinated for COVID-19. The dependent variable indicates vaccination status, with 69.54% fully vaccinated and 30.46% not vaccinated, corresponding to an odds ratio of 5.212062 (0.6954/0.3046 * 0.6954/0.3046) under the alternative hypothesis. We used a two-tailed Z-test with an alpha error probability of 0.05 and a desired power of 0.95. We incorporated a binomial distribution for an additional independent variable with parameter π equals to 0.1. We accounted for multicollinearity with a Pseudo R2 of 0.2 for other predictors, using information from the multivariate model in Table 3. Applying the large sample approximation and the Demidenko (2007) test procedure without variance correction, the analysis determined a critical z-value of 1.9599640 and a required total sample size of 313 to achieve an actual power of 0.9502377 [57]. These findings ensure our study with a sample of 5,240 respondents is adequately powered to detect meaningful associations between our independent variables and vaccination status.

The following section presents the results of our empirical findings. We illustrate a series of univariate, bivariate, and multivariate statistical findings to test our hypotheses. (See Supporting Information files (S1 and S2 Databases; S3 and S4 Codes) for data files and code).

## Main results

Tables 1 and 2 illustrate bivariate analyses between COVID-19 vaccination (dependent variable) and the independent variables: race/ethnicity, sex, age groups, education groups, political party, religious groups, and main source of information about COVID-19 vaccine. Specifically, Table 1 illustrates frequency distributions for the variables featured in the main analyses. This table also contains cross tabulations between COVID-19 vaccination and each of the independent variables. Further, Table 2 shows seven unadjusted logistic regression models (one model for each independent variable).

In addition to the main independent variable, COVID-19 vaccination status, we collected several demographic characteristics of respondents, including race/ethnicity, sex, age, and educational attainment. First, at the time of data collection in 2023, 69.54% of respondents were fully vaccinated and 30.46% of respondents were unvaccinated or partially vaccinated (Table 1). Moreover, the majority of respondents were non-Hispanic White. Our sample was comparably representative of males and females. For the age variable, there were relatively similar percentages of respondents falling into age groups between 18 and 64. In terms of educational achievement, most respondents reported either a high school diploma, some college, or a bachelor’s degree. Respondents were also asked to report their political party, including the strength of their association with that party, and religious identification. The sample distribution shows a substantial range in political party affiliations and strength. For religious identification, most respondents identified as some form of Protestant or as “nothing in particular. Lastly, the majority of respondents reported either the Centers for Disease Control and Prevention (CDC) or other health sources (i.e., the FDA, as well as respondent’s health insurer, hospital system, local health, nurses, pharmacists, primary care, state health, and online medical) as their main source of information about vaccines.

According to the distribution of respondents by COVID-19 vaccination status (Table 1) and the unadjusted models (Table 2), except for age group, fully vaccination patterns by sociodemographic characteristics follow Hypothesis 1: More vulnerable populations are less likely to be vaccinated for COVID-19. In each ethnoracial group, the majority of respondents were fully vaccinated, but there were some important variations across ethnoracial groups. African American respondents had the lowest percentage of being fully vaccinated (63.01%) while Asians had the highest percentage of being fully vaccinated (85.41%). According to Table 2, the unadjusted model with the ethnoracial measure as the only independent variable shows that in comparison to White respondents, African Americans have a 28.1% reduction in their odds [(0.719–1)*100; p < .01] of reporting being fully vaccinated and “Other” respondents have a 22.9% reduction [(0.771–1)*100; p < .1], while Asians’ odds of being fully vaccinated increase by 147.1% [(2.471-1)*100; p < .01]. More males (73.72%) than females (65.76%) reported being fully vaccinated, which parallels the unadjusted model in Table 2 because females’ odds of being fully vaccinated are 31.6% lower relative to males [(0.684–1)*100; p < .01]. Lastly, Table 1 illustrates that higher levels of educational attainment were associated with higher likelihood of being fully vaccinated. For example, while 87.99% of respondents with a graduate degree were fully vaccinated, only 56.04% of respondents with a high school diploma reported being fully vaccinated. Similarly, according to Table 2, in comparison to respondents with a high school level of education, all levels above high school have greater odds of being fully vaccinated while the odds for those with less than a high school level of education are reduced almost by half [(0.531–1)*100] (all coefficients are significant at the p < .01-level).

Contrary to Hypothesis 1, older individuals were more likely to be fully vaccinated, which might be a result of the strong public health messages emphasizing the need for these individuals to get vaccinated against COVID-19. The 18–29 age group has the lowest percentage of being fully vaccinated (59.55%) while older age groups, especially the 75 + age group with 85.99%, have notably higher percentages of being fully vaccinated. Shifting over to the unadjusted model in Table 2, with respect to the 30–39 age group, the age groups starting at age 50 or above all have greater odds of being fully vaccinated, while the youngest age group –18–29– have 22.3% odds decrease in being fully vaccinated [(0.777–1)*100; all mentioned coefficients are significant at the p < .01-level].

COVID-19 vaccination status by political parties and religious groups give support to Hypothesis 2: Conservative political background leads to lower COVID-19 vaccinations. Although all groups were more likely to report being fully vaccinated than not, it was to varying degrees. According to the percentage distribution, 86.65% of strong Democrats reported being vaccinated, while 58.54% of strong Republicans reported being vaccinated. For Democrats, stronger party affiliation is associated with higher rates of vaccination, while for Republicans, stronger party affiliation is minimally associated with lower rates of vaccination. Referring to Table 2’s unadjusted model, compared to the reference group –strong Democrat– all other political party categories have lower odds of being fully vaccinated (all coefficients are significant at the p < .01-level). Moreover, for all categories of religious identification, respondents were more likely to report being fully vaccinated than not. However, it is important to note that Evangelical Protestants had one of the smallest percentages to be fully vaccinated against COVID-19 (62.83%) while “Other” has the highest (85.36%). Table 2’s unadjusted model adds proof to the possible influence of religious background. An important note is that this variable about religious groups does not measure the strength of religious beliefs or religiosity. All we can assess is whether religious groups are associated with respondents being fully vaccinated for COVID-19. These results indicate that relative to identifying as an Evangelical Protestant, all categories –except for “nothing in particular” – have greater odds of being fully vaccinated (all coefficients are significant at the p < .01-level).

Lastly, individuals who got information on COVID-19 vaccination from the CDC (83.35%) had the highest percentage of all information categories to be fully vaccinated while respondents who reported that “Other sources” such as religious leaders, social media, and union leaders were their main source of information had the lowest percentage of being fully vaccinated (29.22%). Moreover, Table 2’s unadjusted model for the main source of information shows that relative to reporting the CDC as their main information source, all other sources are associated with substantially lower odds of a respondent being fully vaccinated, especially those who report “Other sources” with a 91.8% decrease in the odds [(0.082–1)*100]. All coefficients are significant at the p < .01-level. These findings are in line with our Hypothesis 3: Relative to individuals who rely on health experts as their main information source, in particular the CDC, individuals who rely on more informal networks are less likely to be fully vaccinated.

Following the previous bivariate analysis of COVID-19 vaccination by independent variables, we estimated a multivariate logistic regression model to elucidate which factors presented the strongest and statistically significant associations with being fully vaccinated (Table 3). The overall statistical significance of the model is underscored by the Pseudo R-squared of 0.174 and the likelihood ratio chi-squared test of 1,120.84, indicating its robust predictive capacity for vaccination status. Examining the odds ratios for individual predictor variables provides nuanced insights into their effects on vaccination likelihood.

Demographic variables exhibit substantial influence on the chances to be fully vaccinated for COVID-19, as illustrated on Table 3. The patterns in the full model in general follow the broad patterns of Table 2’s bivariate analysis. Like in the bivariate analysis, our findings are in line with Hypothesis 1: More vulnerable groups are less likely to be fully vaccinated for COVID-19. However, race/ethnicity groups exhibit interesting patterns related to COVID-19 vaccination that do not entirely fit with this hypothesis. In a multivariate analysis, we would expect that Hispanics have lower levels of vaccination than the White population, because they are a more vulnerable population group. However, our findings indicate that there is no statistically significant difference in the odds of being fully vaccinated among Hispanic compared to White peoples when controlling for all other independent variables. This finding could be related to the Hispanic mortality paradox, i.e., cultural and sociodemographic factors that influence better health behaviors and outcomes among Hispanics, even with their lower socioeconomic status than White people. Asians have a 118.6% increase in their odds of being fully vaccinated for COVID-19 compared to White people, controlled for the other independent variables [(2.186-1)*100; p < .01]. On the other hand, African Americans have roughly a 20% decrease in their odds of being vaccinated relative to White people [(.827-1)*100; p < .1)], which emphasizes another vulnerable situation this group experiences in the population. Unlike in the bivariate analysis, the results for the “Other” ethnoracial category are no longer even marginally significant.

There is additional support for Hypothesis 1 when focusing on the variables “sex” and “education group”. According to the adjusted model, females’ odds of being vaccinated are approximately 25% lower [(.747-1)*100; p < .01], compared to males, suggesting a gender-based discrepancy in vaccination access and decisions. Our findings support existing research on COVID vaccines that has pointed to the gender discrepancy in vaccination rates [58,59]. These studies indicate that, in contrast to men who are more likely to minimize health risk of COVID 19 and believe in conspiratorial theories, women’s vaccine hesitancy is a result of perceived fear of side effects of the vaccine [58]. Moreover, educational attainment manifests as a strong predictor in the adjusted model, with a discernible trend of higher odds of COVID-19 vaccination associated with increased educational levels. For example, compared to people with a high school degree, those with a bachelor’s degree have an 155.1% increase [(2.551-1)*100] in their odds of being fully vaccinated for COVID-19 while those with a graduate degree have a 272.2% increase [(3.722-1)*100]. Both coefficients are significant at the p < .01-level.

Not in line with Hypothesis 1, individuals in the 50–64, 65–74, and 75 + age groups display increased odds of vaccination, all statistically significant at the p < .01-level. For instance, those age 65–74 have a 134.5% increase [(2.345-1)*100] in their odds of being fully vaccinated for COVID-19, compared to those ages 30–39, controlling for the other independent variables. As noted before, the more dangerous nature of the COVID-19 virus on older individuals might have influenced their vaccination status. Lastly, although the direction of the coefficients are comparable to the bivariate analysis, there is no statistically significant difference between ages 18–29 and 30–39 according to the multivariate analysis.

Notably, as expected by Hypothesis 2, political leanings and religious beliefs demonstrate significant associations with vaccination likelihood in Table 3. Our findings underscore the intricate interplay of cultural, political, and social factors in shaping vaccination decisions. As seen in the bivariate analysis, estimates for political parties according to the multivariate analysis indicate that individuals who self-classify as strong Democrats are more likely to be fully vaccinated for COVID-19, significant at the p < .01-level and controlling for the other independent variables. For instance, compared to strong Democrats, the odds for Democrats decrease by 41.7% [(.583-1)*100] to be vaccinated. This likelihood reaches the lowest values for Republicans [a reduction by 70.8%; (.292-1)*100] and strong Republicans [a reduction by 70.3%; (.297-1)*100]. These findings emphasize that political leanings have strong associations with how individuals faced the pandemic, which includes getting vaccinated for COVID-19.

In terms of religious groups, Evangelical Protestants have the lowest chances of being fully vaccinated for COVID-19 among all religious groups. For instance, Catholics are 56.7% more likely [(1.567–1)*100] to be fully vaccinated than Evangelical Protestants, controlling for the other independent variables. The increase in the likelihood reaches 78.5% [(1.785–1)*100] among individuals who fall in the “Other” category and a 65.2% increase [(1.652–1)*100] among Agnostic/Atheist individuals. All mentioned coefficients are significant at the p < .01-level. However, some religious affiliations (“Other Protestants” and nothing in particular) have higher odds of being fully vaccinated than Evangelical Protestants, but their results are not statistically significant. Besides the change in statistical significance for the “Other Protestant” coefficient, the major patterns of religious affiliation in the multivariate analysis are comparable to the results of the bivariate analysis, which suggests that religious affiliation –like political affiliation– each capture distinct phenomena that are strongly correlated with being fully vaccinated.

Findings also indicate strong and statistically significant associations between the main source of information about COVID-19 vaccines and being fully vaccinated. Individuals whose main source of information is the CDC have the highest chances to be fully vaccinated, controlling for the other independent variables (Table 3). Relative to reporting the CDC as the main source of information, the lowest odds ratios of being fully vaccinated are for individuals whose main sources of vaccine information comes from “Other sources” such as religious leaders, social media, and union leaders [a decrease by 88.9%; (.111-1)*100] and it is statistically significant at the p < .01-level. Other interesting findings indicate that the odds for being fully vaccinated among individuals who mainly receive information from their employer or family and friends is reduced by 66.1% [(.339-1)*100; p < .01] compared to the CDC category, while the category with the second-highest odds ratios of being fully vaccinated are those who rely on “Other health sources” as their main source of information. However, the difference between this category and the reference category is statistically significant at the p < .01-level Overall, these findings –which parallel the bivariate results– highlight that reliance on informal networks such as religious leaders, social media, and personal contacts to receive information about vaccines have the lowest effects on individuals being fully vaccinated for COVID-19, as expected by Hypothesis 3.

Table 4 further emphasizes the combined impact of ethnoracial background and main source of information about COVID-19 vaccines by showing the percentage distribution of main sources of information among White, Hispanic, African American, Asian, and “Other” respondents as well as the percentage of those being fully vaccinated by ethnoracial background and main source combination. Across all ethnoracial categories, the most common primary sources of information about vaccines are “Other health sources” (44.86% among Asians to 52.02% among Hispanics) and the CDC (28.83% among Hispanics to 39.42% among Asians). In turn, more than half of respondents who selected one of these sources as their main source of information, across all ethnoracial categories, report being fully vaccinated. However, there are notable discrepancies worth discussing. For example, just as Asians seem to be at a statistically significant advantage in comparison to White respondents in the adjusted model, this advantage is evident in Table 4’s descriptive statistics. 95.89% and 85.54% of Asian respondents who report the CDC or “Other health sources” as their main source, respectively, were fully vaccinated. Alternatively, among respondents who selected the CDC as their main source, African Americans and respondents belonging to other ethnoracial groups had the lowest rates of being fully vaccinated (76.74% and 76.19%, respectively). Similarly, among respondents whose response falls within the category of “Other health sources”, besides Asians, all other groups report full vaccination rates below 70% and African Americans had the lowest percentage (61.31%).

Less common main sources of information for COVID-19 vaccination further highlight differences across ethnoracial groups. For example, employer or family and friends was the third most common category among all groups minus Asians. A considerable percentage of people indicate that their main source of information about COVID-19 vaccines is employer or family and friends: 8.97% among White people and 10.98% among Hispanics and African Americans. For Asians (n = 5; 2.70%) and “Other” race/ethnicity (n = 21; 9.91%) the sample sizes are small. Due to the smaller sample sizes among the latter groups, vaccination rates should be interpreted with caution. As the adjusted model in Table 3 suggests, vaccination rates for this source category are consistently lower across all ethnoracial groups, especially for Asians (20.00%) and African Americans (41.27%). The only ethnoracial group to have a vaccination rate over 50% in this source category is Hispanics (59.21%). Lastly, for White, Hispanics, and African American respondents, “Other sources” (non-health related) are the least commonly reported main source, and the percentage of those being fully vaccinated are especially low among these mentioned ethnoracial groups (14.29% to 27.78%).

Table 5 shows the results of one multivariate logistic regression model that has interaction effects between race/ethnicity and information source for COVID-19 vaccination as a set of dummies. This model also controls for sex, age group, education group, political party, and religious group. The interaction effects in an adjusted model enhance our understanding of certain patterns evident in Table 4. For example, Asian respondents who select the CDC as their main source of information have over a 300% increase [(4.137-1)*100; p < .05) in the odds of being fully vaccinated relative to White people who select the CDC as their main source of information. Moreover, no other ethnoracial group who report the CDC as their main information source has a statistically significant difference relative to White people who selected the CDC. Second, Asians who report some form of “Other health sources” as their main information source are the only ethnoracial group to not have a statistically significant difference in comparison to White respondents who report the CDC as their main source while other groups such as Hispanics and African American who select “Other health sources” as their main information source are at a significant disadvantage. The information category “Other sources” has a similar pattern. Third, for those with employer or family and friends as their main information source, White people’s odds of being fully vaccinated decrease by 68.7% [(0.313–1)*100; p < .01], Hispanics decrease by 43.8% [(0.562–1)*100; p < .05], and African Americans decrease by 74.7% [(0.253–1)*100; p < .01], compared to White respondents who select the CDC as their main information source. Lastly, for the response category “news sources and professional organizations”, there is a range in terms of whether certain ethnoracial groups have significantly lower odds of being fully vaccinated in comparison to White respondents who report the CDC as their main source of information.

Table 6 shows the results for separate multivariate logistic regression models by ethnoracial background. These models show an even more nuanced analysis of each group since running a model by ethnoracial background is equivalent to interacting race/ethnicity with all independent variables. Some categories for Asians were automatically dropped by the statistical software due to small sample size. In this case, the dropped category is added to the reference category. For instance, since the category of Asians with less than high school was dropped, the remaining odds ratios (some college, associate degree, bachelor, and graduate) have to be interpreted as a comparison to less than high school and high school combined.

However, we focus the analysis on findings that were statistically significant for the reported main source of information about COVID-19 vaccination (reference category: CDC). More specifically, given the lack of statistically significant differences in the odds ratios for main source of information for Asian and other race/ethnicity, the written analysis focuses on White, African American, and Hispanic respondents with particular attention to associations between the main explanatory variable and likelihood of being fully vaccinated.

This last set of results highlight multiple similarities among White, Hispanic, and African American respondents. For example, those who report that their employer or family and friends are their main information source have considerably lower chances to be fully vaccinated, compared to those with CDC as their main information source in each race/ethnicity category: a decrease by 67.3% [(0.327–1)*100; p < .01] among White people, 56.3% [(0.437–1)*100; p < .05] among Hispanics, and 62.7% [(0.373–1)*100; p < .01] among African Americans. The response categories “Other health sources” and “Other sources” have similar patterns. For “Other sources”, the evidence is abundantly clear that compared to those with CDC as main information source in each race/ethnicity category, this category is associated with a dramatic decrease in the odds of being fully vaccinated (p < .01 for all three coefficients across the models). The odds decrease by 89.7% [(0.103–1)*100] for White people, 95.4% [(0.046–1)*100] for Hispanics, and 84.4% [(0.156–1)*100] for African Americans. The most notable difference is that among respondents who select news sources and professional organizations as their main source of information, only White respondents showed a statistically significant difference to respondents of the same ethnoracial group who selected the CDC. More specifically, there is a 63.2% decrease in the odds of being fully vaccinated [(0.368–1)*100; p < .01].

## Final considerations and future directions

In developing, introducing, and distributing a new vaccine, experts often work against the pressures exerted by costs and time. Once an effective and accessible vaccine is in place, the major obstacle in its distribution remains the vaccine’s acceptability. Vaccine acceptability has therefore been the leading indicator for the overall success of vaccination programs [60]. In an age where anti-vaccination attitudes and behavior along with misconceptions about vaccination are rampant [13], increasing vaccine uptake becomes an even more challenging task for policy makers. The World Health Organization therefore cites vaccine refusal and hesitancy in its list of top threats to global public health [50]. Providing insights on the factors influencing the behavior towards COVID-19 vaccines, this study proposes that policies geared toward increasing the acceptance rate of vaccines should be tailored to community characteristics, in particular to political and religious beliefs, and race/ethnicity-related characteristics of a community. It further suggests that information sources play a particularly crucial role in tailoring vaccine campaigns to specific communities.

Our results reveal that vulnerable populations of women, African Americans and those who did not have a college degree were less likely to be vaccinated. Moreover, those younger than 50 years of age exhibited lower levels of vaccination compared to those who are older than 50. These findings confirm the Health Belief Model (HBM) that suggests that people are more likely to engage in health-promoting behavior if they believe they are at a greater health risk and that taking appropriate action will reduce that risk [61,62]. It is well documented that older adults are more susceptible to COVID -19 and are also more likely to experience the pandemic severely. Data indicates that 3 out of 4 deaths caused by COVID-19 are deaths of older adults [63]. Thus, the benefits of vaccination outweigh the barriers to action for older adults. By presenting that old age drives vaccine uptake, our findings indicate that HBM is a useful model for understanding why older adults are more likely to get vaccinated. The study thus reaffirms existing proposals [61] that suggest that public health messaging should highlight the risks that arise from the pandemic and the benefits of vaccination in preventing these risks.

At the same time our findings challenge those who made claims against significant racial or ethnic differences in attitudes toward COVID-19 [64]. Specifically, while for all ethnoracial groups, most of the respondents were fully vaccinated, according to the adjusted model, Asians had a statistically significant increase in their odds of being fully vaccinated compared to Whites, and African Americans had a substantial decrease in their odds of being fully vaccinated in relation to Whites. The study thus identified women, African Americans, those who have less access to education, and those who are younger than 50 as communities that are in need of community tailored vaccine programs that speak to each community’s unique conditions and vulnerabilities. It suggests that public health messages that promote vaccination should be tailored to these groups’ specific beliefs regarding risks.

In line with much of existing research [39,43] we found that Democrats, especially strong Democrats, were more likely to be vaccinated compared to Republicans. These results emphasized the strong association between political identity and vaccine behavior during the COVID-19 pandemic. They further pointed at the importance of recognizing political polarization as an impediment to the success of vaccination campaigns. Sociologists have previously shown that in politically polarized environments an important strategy in mobilizing people behind a campaign is depoliticizing the debate through using scientific frames [65]. When there is political polarization over scientific conclusions such as the need to get the COVID-19 vaccine that strategy however becomes harder to implement. One way to deal with this barrier would be, when shaping campaigns, health authorities and policy makers should recognize and utilize community specific values and norms.

During the COVID-19 pandemic we have seen that some highly religious groups ascribe spiritual forces, both demonic and divine, to the pandemic and the development of vaccines [66]. While those that had demonic vaccine attributions had lower vaccinations rates and greater vaccine hesitancy, those that attributed the development of a vaccine to divine forces had higher vaccine rates for themselves and their children, more positive attitudes towards vaccines, and were more likely to see COVID-19 as a threat. They tended to view vaccines as a way to love their neighbors, and thus live out their religious mandates. While this is only one example, it does show the importance of recognizing community-specific systems of values and how governments can leverage prevailing norms of behavior to promote vaccination.

Our own findings on information sources suggest that another major way of overcoming the rampant political polarization in American society regarding science and vaccine uptakes would be the establishment of alliances with primary information sources in targeted communities. Our findings indicate strong and statistically significant associations between the main source of information about COVID-19 vaccines and being fully vaccinated. Specifically, those individuals whose main sources of vaccine information were informal sources such as employers, family and friends, religious leaders, social media, and union leaders were less likely to report being fully vaccinated. In contrast, those who followed health experts such as the CDC, FDA, hospital system websites, and primary care providers for information on COVID-19 were more likely to get vaccinated. These findings assert that the mediator of vaccination campaigns is at least as important as the message of the vaccination campaign. To diminish misperceptions and generate trust to vaccination programs, we need greater collaboration between the CDC and community specific sources of information. These efforts may involve partnering with individuals that those in more niche, potentially anti-vaccine populations trust [52]. These could include faith leaders, but also doctors and trusted health professionals.

Overall, identifying primary sources of information in given communities, increasing the partnerships and frequency of communication between the CDC and community specific information sources, and tailoring vaccination campaigns to the needs and perceptions of vulnerable communities emerge from our findings as three major strategies in motivating vaccine-hesitant individuals towards vaccine acceptance. Acknowledging and addressing these factors is imperative for the development of targeted public health interventions aimed at promoting widespread COVID-19 vaccination access and acceptance.

Communication strategies that target behavioral change are at the center of contemporary community-based public health interventions [67]. One study [68] specifically emphasizes evidence-based communication strategies, noting that they are essential when dealing with community members in order to control COVID-19 vaccine-related misinformation and to ensure large public benefits. Building on this insight, our study offers a foundation for tailoring vaccine campaigns to communities. Based on our findings, we propose that information sources constitute a primary means of tailored vaccine campaigns. While communication of content is important for vaccine acceptance–and not just through short-term campaigns but also through long-term communicative approaches [69], our results indicate that vaccine campaigns reach relevant audiences only when they are distributed by trusted sources. By highlighting possible mechanisms through which public health messages reach the target populations, we offer a framework that can help tailor communication messages to communities and make them more efficient. These strategies can then increase vaccination uptake, saving lives and valuable resources during the current and future pandemics led by emerging infectious diseases.

A key limitation of our correlational study is that we cannot make any causal inferences as to which factors are driving vaccination behavior. Another limitation is that the data are collected retrospectively and thus rely on respondents’ ability to recall accurately 1) if they have received a COVID-19 vaccination, and if so, 2) did they complete the COVID-19 vaccination schedule. While we do make assumptions about respondents’ abilities to correctly remember their vaccination status, collecting data on vaccination behavior after the peak of the pandemic grants us a fuller picture of people who vaccinated earlier on as vaccines were first released for public use as well later after the initial wave of vaccinations.

Future research should analyze predictors of vaccination by accessing data that may capture vaccination behavior more accurately, such as respondents’ health records. Although this study features a large sample size that speaks to the broad U.S. population as well as a thorough, detailed analysis of several factors that can impact vaccination behavior, an insightful avenue of future research is to reconduct this analysis using another form of vaccination data.

Further, acceptance of a new vaccine is often addressed as a matter of psychological behavior where people feeling fearful, or distrustful towards the vaccine show unwillingness toward taking the vaccine [70]. Indeed, during the COVID-19 pandemic, scholars have shown that public trust in the vaccine to be safe and effective after administration was the strongest forecaster of COVID-19 vaccine uptake intention. While emotions certainly play a key role in motivating one’s actions towards a new vaccine, social sciences remind us that emotions are socially produced [71–73]. The framing of information and how we receive the information play key roles in giving direction to our emotions vis-a-vis vaccines. Our findings also call for further scholarship that understands emotions toward vaccines in the context of individuals’ community memberships. Afterall, in tailoring vaccination campaigns to specific vulnerabilities and needs of communities the task ahead will be enhancing community members’ trust to the vaccine, its developers and distributors.

## Supporting information

S1 Database Database in excel. (XLSX)

S2 Database Database in DTA (Stata). (DTA)

S3 Codes Codes in TXT. (TXT)

S4 Codes Codes in DO (Stata). (DO)

## References

[1] Baden LR, El Sahly HM, Essink B, Kotloff K, Frey S, Novak R, et al. Efficacy and Safety of the mRNA-1273 SARS-CoV-2 Vaccine. N Engl J Med. 2021;384(5):403–16. doi: 10.1056/NEJMoa2035389 33378609 PMC7787219

[2] Polack FP, Thomas SJ, Kitchin N, Absalon J, Gurtman A, Lockhart S, et al. Safety and Efficacy of the BNT162b2 mRNA Covid-19 Vaccine. N Engl J Med. 2020;383(27):2603–15. doi: 10.1056/NEJMoa2034577 33301246 PMC7745181

[3] Mallapaty S. Can COVID vaccines stop transmission? Scientists race to find answers. Nature. 2021:10.1038/d41586-021-00450-z. doi: 10.1038/d41586-021-00450-z 33608683

[4] Roy DN, Biswas M, Islam E, Azam MS. Potential factors influencing COVID-19 vaccine acceptance and hesitancy: A systematic review. PLoS One. 2022;17(3):e0265496. doi: 10.1371/journal.pone.0265496 35320309 PMC8942251

[5] Ndugga N, Hill L, Artiga S, Haldar S. Latest data on COVID-19 vaccinations by Race/Ethnicity [Internet]. KFF. 2022 Jul 14. Available from: https://www.kff.org/coronavirus-covid-19/issue-brief/latest-data-on-covid-19-vaccinations-race-ethnicity/

[6] Artiga S. Growing gaps in COVID-19 vaccinations among Hispanic people [Internet]. KFF. 2021 Feb 22. Available from: https://www.kff.org/policy-watch/growing-gaps-covid-19-vaccinations-hispanic-people/

[7] Synnott CK. Managing college students’ COVID-19 vaccine hesitancy [Internet]. University Business. 2021 Jan 8. Available from: https://universitybusiness.com/managing-college-students-covid-19-vaccine-hesitancy/

[8] World Health Organization. Report of the SAGE Working Group on Vaccine Hesitancy [Internet]. 2014 Nov [cited 2024 Jul 6]. p. 1–64. Available from: https://www.asset-scienceinsociety.eu/pages/report-sage-working-group-vaccine-hesitancy

[9] Cooper S, Schmidt BM, Sambala EZ, Swartz A, Colvin CJ, Leon N, et al. Factors that influence parents’ and informal caregivers’ acceptance of routine childhood vaccination: a qualitative evidence synthesis. Cochrane Database Syst Rev [Internet]. 2019 Feb 18 [cited 2019 Sep 13]; Available from: https://www.cochrane.org/CD013265/EPOC_factors-influence-parents-and-informal-caregivers-acceptance-routine-childhood-vaccination 10.1002/14651858.CD013265.pub2 PMC8550333 34706066

[10] MacDonald NE, SAGE Working Group on Vaccine Hesitancy. Vaccine hesitancy: Definition, scope and determinants. Vaccine. 2015;33(34):4161–4. doi: 10.1016/j.vaccine.2015.04.036 25896383

[11] Dubé E, Laberge C, Guay M, Bramadat P, Roy R, Bettinger J. Vaccine hesitancy: an overview. Hum Vaccin Immunother. 2013;9(8):1763–73. doi: 10.4161/hv.24657 23584253 PMC3906279

[12] Larson HJ, Jarrett C, Eckersberger E, Smith DMD, Paterson P. Understanding vaccine hesitancy around vaccines and vaccination from a global perspective: a systematic review of published literature, 2007-2012. Vaccine. 2014;32(19):2150–9. doi: 10.1016/j.vaccine.2014.01.081 24598724

[13] Xiao X, Wong RM. Vaccine hesitancy and perceived behavioral control: A meta-analysis. Vaccine. 2020;38(33):5131–8. doi: 10.1016/j.vaccine.2020.04.076 32409135

[14] Nichter M. Vaccinations in the Third World: a consideration of community demand. Soc Sci Med. 1995;41(5):617–32. doi: 10.1016/0277-9536(95)00034-5 7502096

[15] Streefland PH. Public doubts about vaccination safety and resistance against vaccination. Health Policy. 2001;55(3):159–72. doi: 10.1016/s0168-8510(00)00132-9 11164965

[16] Reich JA. Neoliberal Mothering and Vaccine Refusal. Gender & Society. 2014;28(5):679–704. doi: 10.1177/0891243214532711

[17] Berezin M, Eads A. Risk is for the rich? Childhood vaccination resistance and a Culture of Health. Soc Sci Med. 2016;165:233–45. doi: 10.1016/j.socscimed.2016.07.009 27499069

[18] Dunn AG, Surian D, Leask J, Dey A, Mandl KD, Coiera E. Mapping information exposure on social media to explain differences in HPV vaccine coverage in the United States. Vaccine. 2017;35(23):3033–40. doi: 10.1016/j.vaccine.2017.04.060 28461067

[19] Myers LB, Goodwin R. Determinants of adults’ intention to vaccinate against pandemic swine flu. BMC Public Health. 2011;11(1):15. doi: 10.1186/1471-2458-11-15 21211000 PMC3024930

[20] Dimala CA, Kadia BM, Nji MAM, Bechem NN. Factors associated with measles resurgence in the United States in the post-elimination era. Sci Rep. 2021;11(1):51. doi: 10.1038/s41598-020-80214-3 33420153 PMC7794463

[21] COVID-19 Provisional counts - Health disparities [Internet]. Available from: https://www.cdc.gov/nchs/nvss/vsrr/covid19/health_disparities.htm

[22] Agarwal R, Dugas M, Ramaprasad J, Luo J, Li G, Gao GG. Socioeconomic privilege and political ideology are associated with racial disparity in COVID-19 vaccination. Proc Natl Acad Sci U S A. 2021;118(33):e2107873118. doi: 10.1073/pnas.2107873118 34326130 PMC8379950

[23] Bagasra AB, Doan S, Allen CT. Racial differences in institutional trust and COVID-19 vaccine hesitancy and refusal. BMC Public Health. 2021;21(1):2104. doi: 10.1186/s12889-021-12195-5 34789205 PMC8595958

[24] Colvin KM, Camara KS, Adams LS, Sarpong AP, Fuller DG, Peck SE, et al. Profiles of COVID-19 vaccine hesitancy by race and ethnicity in eastern Pennsylvania. PLoS One. 2023;18(2):e0280245. doi: 10.1371/journal.pone.0280245 36745588 PMC9901750

[25] Heiman SL, Hirt ER, Isch C, Brinkworth JF, Cronk L, Alcock J, et al. Identities as predictors of vaccine hesitancy during the COVID‐19 pandemic. Journal of Social Issues. 2022;79(2):556–77. doi: 10.1111/josi.12569

[26] Health Concerns From COVID-19 Much Higher Among Hispanics and Blacks Than Whites | Pew Research Center [Internet]. [cited 2024 Jun 4]. Available from: https://www.pewresearch.org/politics/2020/04/14/health-concerns-from-covid-19-much-higher-among-hispanics-and-blacks-than-whites/

[27] Daly M, Robinson E. Willingness to vaccinate against COVID-19 in the U.S.: Representative longitudinal evidence from April to October 2020. Am J Prev Med. 2021 Feb. 10.1016/j.amepre.2021.01.008 PMC7883746 33773862

[28] A meta-analysis of COVID-19 vaccine attitudes and demographic characteristics in the United States - PMC [Internet]. [cited 2024 Jun 4]. Available from: https://www.ncbi.nlm.nih.gov/pmc/articles/PMC8958161/10.1016/j.puhe.2022.03.012 PMC8958161 35486981

[29] Frisco ML, Van Hook J, Thomas KJA. Racial/ethnic and nativity disparities in U.S. Covid-19 vaccination hesitancy during vaccine rollout and factors that explain them. Soc Sci Med. 2022;307:115183. doi: 10.1016/j.socscimed.2022.115183 35843179 PMC9242888

[30] Khubchandani J, Sharma S, Price JH, Wiblishauser MJ, Sharma M, Webb FJ. COVID-19 Vaccination Hesitancy in the United States: A Rapid National Assessment. J Community Health. 2021;46(2):270–7. 33389421 10.1007/s10900-020-00958-x PMC7778842

[31] Kricorian K, Turner K. COVID-19 Vaccine Acceptance and Beliefs among Black and Hispanic Americans. PLoS One. 2021;16(8):e0256122. doi: 10.1371/journal.pone.0256122 34428216 PMC8384224

[32] Nguyen LH, Joshi AD, Drew DA, Merino J, Ma W, Lo C-H, et al. Self-reported COVID-19 vaccine hesitancy and uptake among participants from different racial and ethnic groups in the United States and United Kingdom. Nat Commun. 2022;13(1):636. doi: 10.1038/s41467-022-28200-3 35105869 PMC8807721

[33] Momplaisir F, Haynes N, Nkwihoreze H, Nelson M, Werner RM, Jemmott J. Understanding Drivers of Coronavirus Disease 2019 Vaccine Hesitancy Among Blacks. Clin Infect Dis. 2021;73(10):1784–9. doi: 10.1093/cid/ciab102 33560346 PMC7929035

[34] COVID‐19 Disparities and Vaccine Hesitancy in Black Americans: What Ethical Lessons Can Be Learned? - Restrepo - 2022 - Otolaryngology–Head and Neck Surgery - Wiley Online Library [Internet]. [cited 2024 Jun 4]. Available from: https://aao-hnsfjournals.onlinelibrary.wiley.com/doi/10.1177/01945998211065410 10.1177/01945998211065410 34905417

[35] Callaghan T, Moghtaderi A, Lueck JA, Hotez P, Strych U, Dor A, et al. Correlates and disparities of intention to vaccinate against COVID-19. Soc Sci Med. 2021;272:113638. doi: 10.1016/j.socscimed.2020.113638 33414032 PMC7834845

[36] Khubchandani J, Macias Y. COVID-19 vaccination hesitancy in Hispanics and African-Americans: A review and recommendations for practice. Brain Behav Immun Health. 2021;15:100277. doi: 10.1016/j.bbih.2021.100277 34036287 PMC8137342

[37] Malik AA, McFadden SM, Elharake J, Omer SB. Determinants of COVID-19 vaccine acceptance in the US. EClinicalMedicine. 2020;26:100495. doi: 10.1016/j.eclinm.2020.100495 32838242 PMC7423333

[38] Sileo KM, Hirani IM, Luttinen RL, Hayward M, Fleming PJ. A Scoping Review on Gender/Sex Differences in COVID-19 Vaccine Intentions and Uptake in the United States. Am J Health Promot. 2024;38(2):242–74. doi: 10.1177/08901171231200778 37847250 PMC10802093

[39] Funk C, Tyson A, Kennedy B, Pasuini G. Americans’ largely positive views of childhood vaccines hold steady. Pew Research Center. 2023. Available from: https://www.pewresearch.org/science/2023/05/16/americans-largely-positive-views-of-childhood-vaccines-hold-steady/

[40] Moore JX, Gilbert KL, Lively KL, Laurent C, Chawla R, Li C, et al. Correlates of COVID-19 Vaccine Hesitancy among a Community Sample of African Americans Living in the Southern United States. Vaccines (Basel). 2021;9(8):879. doi: 10.3390/vaccines9080879 34452004 PMC8402307

[41] Pasquini G, Saks E. Partisan differences are common in the lessons Americans take away from COVID-19. Pew Research Center. 2022. Available from: https://www.pewresearch.org/short-reads/2022/09/06/partisan-differences-are-common-in-the-lessons-americans-take-away-from-covid-19/

[42] Corcoran KE, Scheitle CP, DiGregorio BD. Christian nationalism and COVID-19 vaccine hesitancy and uptake. Vaccine. 2021;39(45):6614–21. doi: 10.1016/j.vaccine.2021.09.074 34629205 PMC8489517

[43] Cowan SK, Mark N, Reich JA. COVID-19 vaccine hesitancy is the new terrain for political division among Americans. Socius. 2021;7:237802312110236.

[44] Alemi F, Lee KH. Impact of Political Leaning on COVID-19 Vaccine Hesitancy: A Network-Based Multiple Mediation Analysis. Cureus. 2023;15(8):e43232. doi: 10.7759/cureus.43232 37692573 PMC10491458

[45] Albrecht D. Vaccination, politics and COVID-19 impacts. BMC Public Health. 2022;22(1):96. doi: 10.1186/s12889-021-12432-x 35031053 PMC8758893

[46] Jung Y, Lee S. Trump vs. the GOP: Political Determinants of COVID-19 Vaccination. Polit Behav. 2023;46(3):1511–33. doi: 10.1007/s11109-023-09882-x

[47] Travis J, Harris S, Fadel T, Webb G. Identifying the determinants of COVID-19 preventative behaviors and vaccine intentions among South Carolina residents. PLoS One. 2021;16(8):e0256178. doi: 10.1371/journal.pone.0256178 34432817 PMC8386860

[48] Whitehead AL, Perry SL. How Culture Wars Delay Herd Immunity: Christian Nationalism and Anti-vaccine Attitudes. Socius: Sociological Research for a Dynamic World. 2020;6. doi: 10.1177/2378023120977727

[49] Jurkowitz M, Mitchell A. Americans who relied most on Trump for COVID-19 news among least likely to be vaccinated. Pew Research Center. 2021. Available from: https://www.pewresearch.org/short-reads/2021/09/23/americans-who-relied-most-on-trump-for-covid-19-news-among-least-likely-to-be-vaccinated/

[50] Hou Z, Tong Y, Du F, Lu L, Zhao S, Yu K, et al. Assessing COVID-19 Vaccine Hesitancy, Confidence, and Public Engagement: A Global Social Listening Study. J Med Internet Res. 2021;23(6):e27632. doi: 10.2196/27632 34061757 PMC8202656

[51] Hornsey MJ, Edwards M, Lobera J, Díaz-Catalán C, Barlow FK. Resolving the small-pockets problem helps clarify the role of education and political ideology in shaping vaccine scepticism. Br J Psychol. 2021;112(4):992–1011. doi: 10.1111/bjop.12500 33715151

[52] Guidry JPD, Miller CA, Perrin PB, Laestadius LI, Zurlo G, Savage MW, et al. Between Healthcare Practitioners and Clergy: Evangelicals and COVID-19 Vaccine Hesitancy. Int J Environ Res Public Health. 2022;19(17):11120. doi: 10.3390/ijerph191711120 36078836 PMC9518019

[53] Fisher KA, Nguyen N, Crawford S, Fouayzi H, Singh S, Mazor KM. Preferences for COVID-19 vaccination information and location: Associations with vaccine hesitancy, race and ethnicity. Vaccine. 2021;39(45):6591–4. doi: 10.1016/j.vaccine.2021.09.058 34629210 PMC8463309

[54] Meltzer GY, Chang VW, Lieff SA, Grivel MM, Yang LH, Des Jarlais DC. Behavioral Correlates of COVID-19 Worry: Stigma, Knowledge, and News Source. Int J Environ Res Public Health. 2021;18(21):11436. doi: 10.3390/ijerph182111436 34769952 PMC8583421

[55] Prime Panels [Internet]. Available from: https://www.cloudresearch.com/products/prime-panels/?ga_ref=products

[56] Chandler J, Rosenzweig C, Moss AJ, Robinson J, Litman L. Online panels in social science research: Expanding sampling methods beyond Mechanical Turk. Behav Res Methods. 2019;51(5):2022–38. doi: 10.3758/s13428-019-01273-7 31512174 PMC6797699

[57] Demidenko E. Sample size determination for logistic regression revisited. Stat Med. 2007;26(18):3385–97. doi: 10.1002/sim.2771 17149799

[58] Morales DX, Beltran TF, Morales SA. Gender, socioeconomic status, and COVID-19 vaccine hesitancy in the US: An intersectionality approach. Sociol Health Illn. 2022;44(6):953–71. doi: 10.1111/1467-9566.13474 35500003 PMC9348198

[59] Toshkov D Explaining the gender gap in COVID-19 vaccination attitudes. European Journal of Public Health. 2023;33(3):490–5. 37178211 10.1093/eurpub/ckad052 PMC10234652

[60] Mahoney RT, Krattiger A, Clemens JD, Curtiss R 3rd. The introduction of new vaccines into developing countries. IV: Global Access Strategies. Vaccine. 2007;25(20):4003–11. doi: 10.1016/j.vaccine.2007.02.047 17363119

[61] Subedi S, Leal Filho W, Adedeji A. An assessment of the health belief model (HBM) properties as predictors of COVID-19 preventive behaviour. J Public Health (Berl). 2023. doi: 10.1007/s10389-023-02109-7

[62] Jones CL, Jensen JD, Scherr CL, Brown NR, Christy K, Weaver J. The Health Belief Model as an explanatory framework in communication research: exploring parallel, serial, and moderated mediation. Health Commun. 2015;30(6):566–76. doi: 10.1080/10410236.2013.873363 25010519 PMC4530978

[63] Graham J, News KH. Thousands of seniors are still dying of Covid-19. Do we not care anymore? CNN; 2024. Available from: https://www.cnn.com/2024/02/08/health/aging-discrimation-kff-partner-wellness/index.html

[64] Scheitle CP, Corcoran KE. COVID-19 Skepticism in Relation to Other Forms of Science Skepticism. Socius: Sociological Research for a Dynamic World. 2021;7. doi: 10.1177/23780231211049841

[65] Iturriaga N. Exhuming Violent Histories: Forensics, Memory, and Rewriting Spain’s Past. Columbia University Press; 2022.

[66] Exline JJ, Pait KC, Wilt JA, Schutt WA. Demonic and Divine Attributions around COVID-19 Vaccines: Links with Vaccine Attitudes and Behaviors, QAnon and Conspiracy Beliefs, Anger, Spiritual Struggles, Religious and Political Variables, and Supernatural and Apocalyptic Beliefs. Religions. 2022;13(6):519. doi: 10.3390/rel13060519

[67] Agency for Healthcare Research and Quality. Evidence-based practice center systematic review protocol: Communication and dissemination strategies to facilitate the use of health and health care evidence [Internet]. 2012. Available from: https://effectivehealthcare.ahrq.gov/sites/default/files/pdf/medical-evidence-communication_research-protocol.pdf

[68] Rzymski P, Borkowski L, Drąg M, Flisiak R, Jemielity J, Krajewski J, et al. The Strategies to Support the COVID-19 Vaccination with Evidence-Based Communication and Tackling Misinformation. Vaccines (Basel). 2021;9(2):109. doi: 10.3390/vaccines9020109 33535716 PMC7912910

[69] Arede M, Bravo-Araya M, Bouchard É, Singh Gill G, Plajer V, Shehraj A, et al. Combating Vaccine Hesitancy: Teaching the Next Generation to Navigate Through the Post Truth Era. Front Public Health [Internet]. 2019 Jan 14 [cited 2024 Jun 4];6. Available from: https://www.frontiersin.org/journals/public-health/articles/10.3389/fpubh.2018.00381/full 10.3389/fpubh.2018.00381 PMC6339919 30693276

[70] Quinn SC, Jamison AM, An J, Hancock GR, Freimuth VS. Measuring vaccine hesitancy, confidence, trust and flu vaccine uptake: Results of a national survey of White and African American adults. Vaccine. 2019;37(9):1168–73. doi: 10.1016/j.vaccine.2019.01.033 30709722

[71] Bericat E. The sociology of emotions: Four decades of progress. Current Sociology. 2015;64(3):491–513. doi: 10.1177/0011392115588355

[72] Berezin M. Secure States: Towards a Political Sociology of Emotion. The Sociological Review. 2002;50(2_suppl):33–52. doi: 10.1111/j.1467-954x.2002.tb03590.x

[73] Hochschild AR. The sociology of emotion as a way of seeing. In: Emotions in Social Life. Routledge; 1997. doi: 10.4324/9780203437452-10

