# Supplementary material for: A comprehensive analysis of COVID-19 vaccination behavior: The influence of religion, information sources, political leanings, and demographic factors
Source: PLoS One. 2025 May 21;20(5):e0323815. doi: 10.1371/journal.pone.0323815 (PMC12094743; doi:10.1371/journal.pone.0323815)
Supplement: Attachment — Submitted filename: Response_to_Reviewers.docx [file pone.0323815.s006.docx]

We thank the editor and the reviewers for providing us with an opportunity to revise and resubmit our manuscript. As we rewrote the manuscript to bring it in line with the encouraging and constructive comments provided by the reviewers, we made sure that the manuscript and the supporting information files meet formatting requirements. Through the revisions made in the introduction and theory sections we established a clear rationale for the study, presented the motivation behind the study and explained what unique perspective emerges from studying this nationally representative dataset. We also included four new tables in the analysis section to present the new analyses. The methods section has been revised to provide more detail on how respondents were selected, consent was gathered, and the data were collected. We also conducted a power analysis to present that the current sample is sufficiently powered for the analyses in this study. Finally, to strengthen the discussion section we included discussions on the Health Belief Model and social norms and removed the earlier emphasis on de/politicization. Please see below for our detailed discussion of the changes that we made on the manuscript. Our responses to reviewer comments are italicized and marked in red. Once again, we would like to express our gratitude to the editor and the reviewers for giving us the opportunity to revise and resubmit our manuscript. We look forward to hearing back from you.

**Journal requirements**

When submitting your revision, we need you to address these additional requirements.

1. Please ensure that your manuscript meets PLOS ONE's style requirements, including those for file naming. The PLOS ONE style templates can be found at

[https://journals.plos.org/plosone/s/file?id=wjVg/PLOSOne_formatting_sample_main_body.pdf](https://urldefense.com/v3/__https://journals.plos.org/plosone/s/file?id=wjVg*PLOSOne_formatting_sample_main_body.pdf__;Lw!!KwNVnqRv!E0aBmpcaImBBb8GIT6mSPygsI1HtJcnLvoi8EiN654xMutpGGHjX9HjeJTRpADDCvk-tof9IBK_G$) and

[https://journals.plos.org/plosone/s/file?id=ba62/PLOSOne_formatting_sample_title_authors_affiliations.pdf](https://urldefense.com/v3/__https://c05y1x9s.r.us-east-2.awstrack.me/L0/https:*2F*2Fjournals.plos.org*2Fplosone*2Fs*2Ffile*3Fid=ba62*2FPLOSOne_formatting_sample_title_authors_affiliations.pdf/1/010f0192b550eeb0-94ff3d77-5b8c-47f1-b966-b603660e1793-000000/G8W8IAHWBXiIthVAEO7ytRETqnU=182__;JSUlJSUlJQ!!KwNVnqRv!CHqc5sgUgy3UwgDt5Jitcf__7LZvhIWdrzEetXj0PYWzm64TAND3wMJjizH_KPlbyFiahmtLUbiamL0$)

The manuscript has been revised to meet style requirements.

2. Please amend your list of authors on the manuscript to ensure that each author is linked to an affiliation. Authors’ affiliations should reflect the institution where the work was done (if authors moved subsequently, you can also list the new affiliation stating “current affiliation:….” as necessary).

The list of authors on the manuscript has been amended to ensure that each author is linked to an affiliation.

3. Please include a caption for table 1 and 2.

Thank you for highlighting this. All tables have been updated to have a caption.

4. We note that the grant information you provided in the ‘Funding Information’ and ‘Financial Disclosure’ sections do not match.

When you resubmit, please ensure that you provide the correct grant numbers for the awards you received for your study in the ‘Funding Information’ section.

We updated the “Funding Information” section to provide the correct grant number for the T3 Grant that we have received from Texas A&M University. The second award that we received from Texas A&M University, namely the Innovation Grant, does not have an award number. The Grant was awarded to us as a team and the system does not allow us to choose us all as grant recipients. For these reasons, we have not been able to enter a grant number and a recipient name.

5. Please amend either the abstract on the online submission form (via Edit Submission) or the abstract in the manuscript so that they are identical.

The abstracts have been amended so that they are identical.

6. We are unable to open your Supporting Information file [Codes_in_Stata.do and Database_in_Stata.dta]. Please kindly revise as necessary and re-upload.

Supporting Information files have been updated. In this round of submission we included the database in two different formats (S1_Database_in_Excel and S2_Database_in_Stata). We also included the codes for Stata in two different formats (S3_Codes_in_TXT and S4_Codes_in_DO files).

7. Please include captions for your Supporting Information files at the end of your manuscript, and update any in-text citations to match accordingly. Please see our Supporting Information guidelines for more information: [http://journals.plos.org/plosone/s/supporting-information](https://urldefense.com/v3/__http://journals.plos.org/plosone/s/supporting-information__;!!KwNVnqRv!E0aBmpcaImBBb8GIT6mSPygsI1HtJcnLvoi8EiN654xMutpGGHjX9HjeJTRpADDCvk-toQ4GBaR3$).

Done.

[Note: HTML markup is below. Please do not edit.]

Reviewers' comments:

Reviewer's Responses to Questions

**Comments to the Author**

1. Is the manuscript technically sound, and do the data support the conclusions?

The manuscript must describe a technically sound piece of scientific research with data that supports the conclusions. Experiments must have been conducted rigorously, with appropriate controls, replication, and sample sizes. The conclusions must be drawn appropriately based on the data presented.

Reviewer #1: No

Reviewer #2: No

2. Has the statistical analysis been performed appropriately and rigorously?

Reviewer #1: No

Reviewer #2: N/A

3. Have the authors made all data underlying the findings in their manuscript fully available?

The [PLOS Data policy](https://urldefense.com/v3/__http://c05y1x9s.r.us-east-2.awstrack.me/L0/http:*2F*2Fwww.plosone.org*2Fstatic*2Fpolicies.action*23sharing/1/010f0192b550eeb0-94ff3d77-5b8c-47f1-b966-b603660e1793-000000/PkTcetiv0Na0amhYLlGAj-G7p6E=182__;JSUlJSU!!KwNVnqRv!CHqc5sgUgy3UwgDt5Jitcf__7LZvhIWdrzEetXj0PYWzm64TAND3wMJjizH_KPlbyFiahmtLg4RHn48$) requires authors to make all data underlying the findings described in their manuscript fully available without restriction, with rare exception (please refer to the Data Availability Statement in the manuscript PDF file). The data should be provided as part of the manuscript or its supporting information, or deposited to a public repository. For example, in addition to summary statistics, the data points behind means, medians and variance measures should be available. If there are restrictions on publicly sharing data—e.g. participant privacy or use of data from a third party—those must be specified.

Reviewer #1: Yes

Reviewer #2: Yes

4. Is the manuscript presented in an intelligible fashion and written in standard English?

PLOS ONE does not copyedit accepted manuscripts, so the language in submitted articles must be clear, correct, and unambiguous. Any typographical or grammatical errors should be corrected at revision, so please note any specific errors here.

Reviewer #1: Yes

Reviewer #2: No

5. Review Comments to the Author

Please use the space provided to explain your answers to the questions above. You may also include additional comments for the author, including concerns about dual publication, research ethics, or publication ethics. (Please upload your review as an attachment if it exceeds 20,000 characters)

**Reviewer #1**

We thank the reviewer for their constructive comments. They have much improved our manuscript.

The description of the methodology for conducting the survey is clearly inadequate.
The organization that conducted the survey, Cloud Research, is named but no information is given about the organization or references to other research conducted by/with the organization is given.
That written informed consent was obtained suggests that the survey was done through face to face interviewing, but this is not clear. Mail interviewing might also have been used.
How subjects were selected and contacted is not specified. Was this a household survey?

*We thank the reviewer for their comments about our description of the data. In the “Data and Methods” section we give additional information on Cloud Research, as well as how respondents were selected, consent was gathered, and the data were collected.*

The response rate and the methods for determining the response rate are not presented.

*Please see the second paragraph of the “Data and Methods” section for more detail on the response rate and the methods determining the response rate.*

Table 2 presents bivariate analyses of the independent variables with the vaccination outcome, but the text describes these relationships as “controlling for other variables. Multivariable logistic results are not included in Table 2.
*We updated the language in the manuscript to explicitly state when we are referring to the bivariate analyses (Table 2’s unadjusted models) relative to the main multivariate results (Table 3).*

The data analysis could have included some form of data reduction, such as factor analysis for the many independent variables.
*We appreciate the level of detail and care the reviewer gave to our methodology. After careful consideration, given that the goal of this study is to do a comprehensive analysis of determinants of vaccination behavior, we have decided to keep the independent variables as is given the extant literature.*

While theory is not required for PLoS One papers, the Health Belief Model would seem to fit these data quite well. It would explain why older subjects were more likely to get vaccinated, which is not explained in the current Discussion.

*We thank the reviewer for their constructive suggestions. In accordance with the reviewer’s comments, we incorporated a discussion on the Health Belief Model to the second and third paragraphs of the “Final considerations and Future Directions” section.*

**Reviewer #2**

*We thank the reviewer for the detail and care that they provided for our manuscript. The revisions made in response to their constructive comments have significanlty improved our manuscript.*

Thank you for the opportunity to review this article. While this topic of research is extremely valuable in the development of tailored future vaccination campaigns, especially considering the diverse demographic composition of the United States, the current paper has several limitations and theoretical shortcomings as discussed below.

**First**, the paper in its current state has several grammatical errors and needs to be heavily edited to ensure succinct sentences for scientific clarity. For ex, Page 1 - paragraph 1, there are several punctuations missing (i.e., missing comma in "During the COVID-19 pandemic vaccines emerged as a...")
**Second**, the paper has several instances of poor sentence construction -

*We thank the reviewer for highlighting the grammatical errors and issues of clarity arising from poor sentence structure. The entire manuscript has been copy-edited to correct these issues. Please also see below for changes made on highlighted errors.*

1. "In the course of the pandemic, reports have shown that the vaccines also prevented disease spread suggesting that vaccinated individuals would not transmit the disease." This can be changed to "Medical reports published during the pandemic established the role of vaccines in limiting the spread of the virus").

*Corrected.*

2. Page 2, "Recognizing that
factors influencing vaccine behavior vary depending on time, place, and vaccines [9, 10, 11], researchers trace the variation in vaccine acceptance to historical, socio-cultural, or political experiences of communities with vaccine administrators, to individual and group level perceptions of the vaccine, to the characteristics of the vaccine and vaccination process." Or is a misplaced word in this sentence and overall this is a difficult sentence to read and clearly understand.

*The sentence has been revised to address the reviewer's comments.*

3. Page 3, paragraph 2's last sentence refers to something called a "common's decision", are the authors referring to individual vaccine decisions being impacted by social norms/a community's opinion? If so, this is not clearly communicated.

*To clarify, “common’s decision” has been changed to “community decision”.*

4. Page 3, last paragraph mentions community characteristics but do not specify what these characteristics are.

*To clarify what community characteristics refer to we’ve added “age, level of education, race/ethnicity, gender” in parentheses.*

5. Page 5, paragraph 2's last sentence reads as, "... women have been getting more vaccines than men", can be re-written to "...women having higher vaccination rates than men."

*The sentence has been corrected.*

6. Page 6, paragraph 2's first sentence is very long and can be broken up into 2 separate sentences to make it more readable.

*The sentence has been revised to make it more readable.*

**Third**, citations have not been consistently included -
*We thank the reviewer for highlighting the missing citations. Please see below for more detail on the citations added.*

1. Page 1 - paragraph 1, "At the same time, with intention to get vaccinated is often associated with higher levels of education...").

*Please see Introduction section, first paragraph, citation [39] for the added citation.*

2. Page 6, last paragraph's first sentence is missing a citation.

*The sentence has been broken down into two separate sentences. Please see the end of the second sentence for the added citation (citation [42]).*

**Fourth**, there are several theoretical and logical gaps in this paper -

1. In its current state, this paper does not establish a clear rationale for how this study adds value to existing research on the topic. It appears that there already exists a lot of data examining COVID-19 vaccination behaviors among various demographic groups in the US as discussed by the authors. How then, is the current study a value addition on the topic?

*We thank the reviewer for their constructive suggestions in highlighting our contribution. In accordance with their comments addressed on item 4 below we added a new paragraph at the end of the theoretical background section. This new paragraph makes a case for this study by highlighting the motivation behind the study and marking its difference from existing studies. The final paragraph of the introduction section has also been revised to highlight that the paper emphasizes the importance of community characteristics on vaccination decisions.*

2. The presentation of a summary of the findings on page 4 seems misplaced since a strong argument for the current study has not yet been presented. One of the findings included on page 4 mentions that "religious beliefs" among Evangelical Protestants are linked to lower vaccination rates. Based on the data collected, as mentioned by the authors, there was no measure of the strength of religious beliefs collected, rather data was collected about what religion one identified with or followed. Therefore, this conclusion is not representative of the data and the findings.

*We thank the reviewer for this comment. We have removed the summary of findings paragraph from the introduction paragraph. In accordance with the comments on item 4 we have instead added a new paragraph to the end of the theoretical background section. Please see our comments under item 4, and the paragraph at the end of the theoretical background section. For the discussion on religious beliefs please also see the revisions made to the text between lines 414 and 423.*

3. Page 6, paragraph 1 presents stats from other studies inconsistently. It would be better to include these statistics consistently for differences between genders, older vs. younger adults, education and income, rather than just reporting them for urban vs. rural areas.

*We agree with the reviewer that the discussion on vaccination in urban vs. rural areas is not connected with the study’s focus on gender, age, education, income and race/ethnicity. To be consistent in our discussion on differences between racial/ethnic groups, genders, older vs. younger adults, education and income we removed the sentence at the end of the first paragraph on page 6.*

4. It would serve well to end the section of the theoretical background by (a) making a case for the current study, (b) including the motivation behind studying this data, (c) whether it adds any unique perspectives to the topic in addition to the research that already exists; (d) if this is a replication of relationships already studied?; (e) how it would aid in the development of vaccination campaigns, etc.

*Along with the reviewer’s constructive suggestions, we added a paragraph at the end of the theoretical background section. This paragraph makes a case for the current study and presents the study’s difference from existing research along with the contribution that arises from the use of a nationally representative sample.*

5. The presentation of hypothesis 1 on page 8 suggests a mediation relationship (i.e., being a member of vulnerable populations leading to the experience of greater obstacles which impacts their likelihood to be vaccinated). However, a mediation relationship was not tested. It would be better to indicate these relationships only when discussing the theoretical background rather than including them in the hypotheses if it is not going to be tested.

*We appreciate the reviewers constructive suggestions. We have carefully considered their comments on the mediation relationship in light of the existing literature which presents that some groups are more vulnerable/disadvantaged than others. Given that the goal of this study is to do a comprehensive analysis of the determinants of vaccination behavior, we have decided to keep the independent variables as is.*

6. The presentation of hypothesis 3 on page 8 mentions "conservative messages against public health messages". However, only reliance on specific information sources was measured in this study. There is no strong argument made to support that certain information sources are always conservative. The authors assume that communication by religious leaders, friends and family to be conservative, which may not always be true. There is no clear presentation of exactly which sources are considered to be conservative vs. liberal, and no strong rationale presented for the assumption either.

*We agree with the reviewer’s comments on hypothesis 3. To address their concerns we have revised the hypothesis. The revised hypothesis states that individuals who follow their personal contacts (family, employer, friends) for information on COVID-19 are less likely to be vaccinated against COVID-19, compared to individuals who follow information sources with expertise on health.*

In the discussion, the authors must also address the critical role of social norms on people's vaccination decisions. It may be almost impossible to change its salience in decision making. In addition to recommending improvements to public vaccination campaigns, the authors should also address how governments can capitalize on the important role on social norms and reduce misinformation among groups by collaborating with religious leaders to communicate accurate information.

*We thank the reviewer for their constructive suggestions. In accordance with their comments, we highlighted the importance of norms in vaccination decisions and vaccination campaigns in the fourth and fifth paragraphs of the “Final considerations and Future Directions” section.*

7. Page 20, paragraph 2 discusses about the depoliticization of vaccination campaigns in the US. Prior to presenting this argument, it would be important to first clearly present research that establishes the politicization of vaccine campaigns in the US. Without it, it only appears to be an assumption by the authors.

*We agree with the reviewer that in the previous version of the manuscript the discussion on depoliticization appeared to be an assumption. To keep the focus of the discussion on political polarization and remove the misplaced emphasis on depoliticization we revised the third sentence of the paragraph. The revised sentence emphasizes that overcoming political polarization matters to the success of vaccination campaigns. The discussion is thus now focused on how polarization could be overcome by recognizing and utilizing community specific values in vaccination campaigns.*

8. Throughout the paper, in discussing hypothesis 3, the authors use messages and messengers (i.e., sources of information) interchangeably. The study only measures reliance/preference of messengers but not the actual content of the messages shared.

*We thank the reviewer for highlighting this issue. We have revised the entire manuscript to remove the emphasis on the role of the content of messages on vaccination decisions.*

9. One significant improvement that would re-position this article's importance would be the examination of interactions between individuals' demographics and their reliance on specific information sources in predicting vaccination behavior. Does the reliance on specific information sources by minorities have differential impact on their vaccination behavior? Are minorities more likely to rely on one or more types of information sources than others? If yes, vaccination campaigns should focus on these sources to deliver accurate and fact-based information to these populations.

*We thank the reviewer for this insightful suggestion. We have added 3 additional tables.*

- *Table 4: a descriptive table to highlight that distribution of the main information source varies by race/ethnicity*
- *Table 5: a table featuring a multivariate logistic regression model which includes a set of dummies for interactions between race/ethnicity and information source and also controls for sex, age group, education group, political party, and religious group.*
- *Table 6: a table with five separate multivariate logistic regression models for each ethnoracial category. These models are the same as interacting race/ethnicity with all independent variables. They provided a more nuanced analysis to each race/ethnicity.*

**Fifth**, there are also some concerns related to the presentation of the methods and results sections -

1. A power analysis supporting that the current sample is sufficiently powered for the analyses in this study is missing.

*Following the reviewer’s suggestion, we conducted a power analysis using G*Power software to determine the required sample size for a logistic regression analysis examining factors associated with our outcome variable. Please see the “Data and Methods” section for further discussion on the power analysis—specifically the 5th paragraph of the section.*

2. On page 10, for most of the measures mentioned, it would be good to include an example item per measure, the format of the response scale (i.e., Likert type scale, etc), response anchors and measures of reliability for any multi-item measures.

*Along with the reviewer’s suggestion to clarify the measures, we mentioned the possible categories for each measure*

3. The analyses presented on page 11 can be included as a separate section titled, "Analyses".

*We appreciate the reviewer’s constructive suggestion on the structure of the paper. After careful consideration, to maintain the existing flow in the text we decided to leave the sections as is.*

4. Some results from the same analysis are presented as odds ratios and others as percentage. In the spirit of consistency, it would be better to present one or the other and to also indicate in the description clearly what the reference group for each of the analyses is and that the differences are significant.

*We have updated the language of the manuscript so that when interpreting coefficients, we discuss the increase/decrease in the odds ratios relative to the reference category as well as mentioning the level of statistical significance.*

5. Page 19, paragraph 1 includes a sentence that appears to be inaccurate, "Other interesting findings indicate that individuals who have nurses for the main information source about vaccines are 68% less likely and those who mainly receive information from family and friends are 68% less likely to be fully vaccinated for COVID-19, compared to the CDC category."
*Given the updated categories for certain variables, this issue has been resolved. The analyses now have 7 age groups (instead of 8); 6 categories of political party (instead of 8); 6 categories for religion (instead of 9); 5 race/ethnicity groups (instead of 6); 5 categories of information source (instead of 18). We made these changes to focus on the main results and to be able to include the interactions of race/ethnicity and information sources. This strategy deals with possible issues of small sample size of some categories. The statement on the nurses has also been dropped as a result.*

6. Page 23, paragraph 2 refers to this as an observational study when in fact it is a correlational study.

*We revised the sentence in accordance with the reviewer’s comments.*

Overall, there is a need and scope for re-positioning the purpose of this article significantly. This data would indeed be a valuable addition to the research on vaccination behavior, especially given the WEIRD problem affecting medical research in the US. However, in my opinion, significant changes to the current draft may be required.

6. PLOS authors have the option to publish the peer review history of their article ([what does this mean?](https://urldefense.com/v3/__https://c05y1x9s.r.us-east-2.awstrack.me/L0/https:*2F*2Fjournals.plos.org*2Fplosone*2Fs*2Feditorial-and-peer-review-process*23loc-peer-review-history/1/010f0192b550eeb0-94ff3d77-5b8c-47f1-b966-b603660e1793-000000/fi0ji3p0CcElHFfFNPCwJmcqvf8=182__;JSUlJSUl!!KwNVnqRv!CHqc5sgUgy3UwgDt5Jitcf__7LZvhIWdrzEetXj0PYWzm64TAND3wMJjizH_KPlbyFiahmtLfmyLAQY$)). If published, this will include your full peer review and any attached files.

If you choose “no”, your identity will remain anonymous but your review may still be made public.


**Do you want your identity to be public for this peer review?** For information about this choice, including consent withdrawal, please see our [Privacy Policy](https://urldefense.com/v3/__https://c05y1x9s.r.us-east-2.awstrack.me/L0/https:*2F*2Fwww.plos.org*2Fprivacy-policy/1/010f0192b550eeb0-94ff3d77-5b8c-47f1-b966-b603660e1793-000000/8qLS5-hXX4exHROzPuZi89nvIgQ=182__;JSUl!!KwNVnqRv!CHqc5sgUgy3UwgDt5Jitcf__7LZvhIWdrzEetXj0PYWzm64TAND3wMJjizH_KPlbyFiahmtLehGEAfY$).

Reviewer #1: No

Reviewer #2: No

[NOTE: If reviewer comments were submitted as an attachment file, they will be attached to this email and accessible via the submission site. Please log into your account, locate the manuscript record, and check for the action link "View Attachments". If this link does not appear, there are no attachment files.]

While revising your submission, please upload your figure files to the Preflight Analysis and Conversion Engine (PACE) digital diagnostic tool, [https://pacev2.apexcovantage.com/](https://urldefense.com/v3/__https://c05y1x9s.r.us-east-2.awstrack.me/L0/https:*2F*2Fpacev2.apexcovantage.com*2F/1/010f0192b550eeb0-94ff3d77-5b8c-47f1-b966-b603660e1793-000000/LLJethkmBaIlwSMTUEM5NgkorsY=182__;JSUl!!KwNVnqRv!CHqc5sgUgy3UwgDt5Jitcf__7LZvhIWdrzEetXj0PYWzm64TAND3wMJjizH_KPlbyFiahmtLlmJayps$). PACE helps ensure that figures meet PLOS requirements. To use PACE, you must first register as a user. Registration is free. Then, login and navigate to the UPLOAD tab, where you will find detailed instructions on how to use the tool. If you encounter any issues or have any questions when using PACE, please email PLOS at figures@plos.org. Please note that Supporting Information files do not need this step.
