# Supplementary material for: A comprehensive analysis of COVID-19 vaccination behavior: The influence of religion, information sources, political leanings, and demographic factors
Source: PLoS One. 2025 May 21;20(5):e0323815. doi: 10.1371/journal.pone.0323815 (PMC12094743; doi:10.1371/journal.pone.0323815)
Supplement: Attachment — Submitted filename: Response to Reviewers.docx [file pone.0323815.s007.docx]

Reviewer #1: The reivewer's comments have been appropriately addressed, with major revisions/improvements made. No further reviewer comments needed.

Thank you for your encouraging review of our manuscript.

Reviewer #3: Thank you for the opportunity to review the manuscript titled 'A Comprehensive Analysis of COVID-19 Vaccination Behavior: The Influence of Religion, Information Sources, Political Leanings, and Demographic Factors'. This is an important and timely study addressing a critical issue with implications for public health communication strategies. The research provides valuable insights into vaccine hesitancy and its determinants, which are crucial for shaping future vaccination campaigns.

We thank the reviewer for their encouraging and helpful comments which significantly improved the presentation of our argument and results.

Below are some comments for revision:

Authors should consider revising the introduction to create a more cohesive structure, combining the overlapping sections into a single, streamlined narrative that flows logically from background to research questions and hypotheses.

After carefully considering the restructuring of these sections to have a more streamlined narrative, we revised both sections. While we kept “Introduction” and “Literature Review” as two separate sections, we made changes to both. Specifically, we shortened the Introduction, moved some information previously included in it to the first and third paragraphs of the Literature Review, and restructured its flow.

In the Methods and Statistical Analysis sections, clearer differentiation is needed. The Methods section should provide more detail on survey design, data collection, and participant demographics, while the Statistical Analysis section should focus on the techniques used, such as regression models.

In accordance with the reviewer’s comments, we subdivided the previous “Data and methods” section into “Methods” and “Statistical analysis” sections to clarify the differentiation.

It would be helpful to elaborate on how the sampling frame and demographic quotas were met, and discuss any limitations in the sampling process. While the absence of a traditional response rate is understandable, a brief discussion on its potential impact on generalizability would be beneficial.

Thank you. We added information about CloudResearch Prime Panels in the new “Methods” section. We also provide information about demographic quotas in this section.

Additionally, a clearer justification for the use of multinomial logistic regression is needed, particularly explaining the decision to collapse partially vaccinated and unvaccinated individuals into one group, and how this choice may affect the interpretation of results.

The models included in our manuscript do not illustrate multinomial logistic regressions. To address the reviewer’s comments, we clarified in the “Statistical analysis” section that we estimated models for different specifications of the dependent variable. However, we included only the logistic models illustrated in the manuscript.

Authors should consider clarifying and streamlining the presentation of demographic data, particularly when describing ethnic/racial groups and vaccination rates, to enhance readability and ensure consistency in how categories are introduced.

The discussion pertaining to the descriptive statistics has been streamlined to highlight the major patterns in the data. Please see changes in the analysis and results sections.

A clearer connection between the unadjusted and multivariate models would also be helpful, especially when discussing the implications of the unadjusted results.

We appreciate this comment and ensured that the main full results are better integrated with the results of the unadjusted models.

Including a brief discussion of sample limitations, such as small sample sizes for certain groups like Asians or "Other" ethnicities, would address potential concerns regarding generalizability.

Thank you. We added information about limitations of small sample sizes for specific race/ethnicity groups and main source of information for COVID-19 vaccination at the end of the “Statistical Analysis” section, related to Table 4 and models on Tables 5 and 6. We also explain this limitation and our approach to interpret the findings in the paragraphs in which we analyze Table 6 in the “Main results” section.

Further interpretation of variables like religion and political party affiliation could enhance the discussion, helping to explore the underlying reasons for the observed differences in vaccination rates.

In the “Main results” section, when we discuss Hypothesis 2, related to religion and political party affiliation, we explain the findings on Tables 2 and 3. We also include this discussion in the “Final considerations” section. We would like to note that in our original submission of this manuscript to the journal, previous reviewers mentioned that we should not attempt to explore underlying reasons for these differences. The argument was that we would need additional variables related to religiosity and political ideology. We made these adjustments in our first resubmission to the journal in response to that first round of review.

In terms of statistical clarity, simplifying the odds ratio interpretations would improve comprehension. For instance, instead of stating "67.3% [(0.327-1)*100; p<.01] less likely among White people," authors should consider revising the phrasing of the odds ratio interpretations to use the actual odds ratios rather than the term "likely," which may cause confusion."

We have updated the language so readers have a clearer understanding of the results.

Finally, clarifying how the dropped categories for Asians due to small sample sizes might affect the results and whether any adjustments were made to address potential bias would strengthen the analysis.

We added information about limitations of small sample sizes for specific race/ethnicity groups and main source of information for COVID-19 vaccination at the end of the "Statistical Analysis” section, related to Table 4 and models on Tables 5 and 6. We also explain this limitation (such as the dropped categories for Asians) and our approach to interpret the findings in the paragraphs in which we analyze Table 6 in the “Main results” section.
